# Unpacking conservation easements’ assessed land use designations and their implications for realizing biodiversity protection

**DOI:** 10.1111/csp2.13130

**Published:** 2024-05-16

**Authors:** Caitlin S. Dyckman, Chris McMahan, Anna Treado Overby, Nakisha Fouch, Scott Ogletree, Stella W. Self, David L. White, Mickey Lauria, Robert F. Baldwin

**Affiliations:** 1 City and Regional Planning Program, School of Architecture, Clemson University, Clemson, South Carolina, USA; 2 School of Mathematics and Statistical Sciences, Clemson University, Clemson, South Carolina, USA; 3 USDA Forest Service, Southern Research Station, Forest Economics and Policy, Forest Science and Assessment Center, Research Triangle Park, North Carolina, USA; 4 Department of Forestry and Environmental Conservation, Clemson University, Clemson, South Carolina, USA; 5 OPENspace Research Centre, Edinburgh College of Art, University of Edinburgh, Edinburgh, UK; 6 Arnold School of Public Health, University of South Carolina, Columbia, South Carolina, USA; 7 Department of Parks, Recreation and Tourism, Clemson University, Watt Family Innovation Center, Clemson, South Carolina, USA

**Keywords:** agriculture, assessed land use designations, biological protection, conservation easements, exurban, open space preservation, residential

## Abstract

Native ecosystem and biodiversity loss from land use conversion into human-modified landscapes are evident in the United States and globally. In addition to public land conservation, there is an increase in private land conservation through conservation easements (CEs) across exurban landscapes. Not every CE was established strictly for biodiversity protection and permitted land uses can increase human modification. No research of which we are aware has examined the actual tax assessor’s land use designations (LUDs) through time. We constructed granular CE datasets (GCED) of CEs and their parcels’ tax assessment LUDs for 1997–2008/2009, based on original data from 12 counties in six US states. Using the GCED, we examined patterns in the LUDs, with implications for land uses that could impact CE biological outcomes. We show that LUDs on exurban private conservation lands were predominately residential and agricultural, with increased residential over time. Critically, the LUDs lack a biological conservation exempt designation/category. There is no consistent trend in association between the primary CE reason and its parcel’s LUD, suggesting that they coincide in some circumstances but in others, the CE may be a response to contravene the LUD. The majority of the first CE reasons are focused on open space preservation, except in some counties where agricultural land uses and agricultural CEs are associated. The economically and human-focused LUD is one of many social factors that should be considered in a classification system for private land conservation and CEs more specifically. These results prompt the land conservation, conservation biology, and environmental planning communities to explore assessed land uses’ impact on biodiversity conservation objectives.

Abstract

## 1 | INTRODUCTION

Biodiversity is rapidly declining in North America and globally (Butchart et al., 2010; Cardinale et al., 2012; Kolbert, 2014), evidenced by the loss of almost 30% of the North American avifauna since 1970 (Rosenberg et al., 2019). This is partially attributed to habitat loss, fragmentation, and other forms of disturbance from land use change (Crooks et al., 2017; Hilty et al., 2006). Over the past 50 years, human demand has “caused unprecedented rates of land and freshwater use” and increased land conversion into agriculture and forestry that have “contributed to increasing net [greenhouse gas] emissions, loss of natural ecosystems…and declining biodiversity” (IPCC, 2019, pp. 2–3). Land conservation is one solution to slow that loss (Hilty et al., 2006) and the most historically prevalent form is public land protection (Protected Areas Database, 2023; IUCN Protected Areas Categories 2004). However there is a growing interest in private lands conservation since findings such as Rissman et al. (2007) suggest that “some or all of the habitat for 85% of federally listed endangered species is found on private land” (p. 710). In 2021, US President Biden’s Administration emphasized private landowners’ voluntary stewardship to help protect 30% of the Nation’s landscape by 2030 (Department of Interior, 2023), mirroring similar global goals.

Conservation easements (CEs) are partial interests in land that have proliferated over the past 40 years across the United States and, more recently, globally (Fishburn et al., 2009; Kamal et al., 2015; Yonavjak & Gartner, 2011). They are deed restrictions that alter a property owner’s ability to use their property for extractive and intensive land use. A governmental entity or a non-governmental not-for-profit entity known as a land trust (LT) holds the severed property right (the CE), enforcing against the property owner if they violate the terms of the CE. LTs use more cost-effective land conservation mechanisms like CEs in lieu of purchasing private parcels, which can be prohibitively expensive (Kiesecker et al., 2007).

According to Parker and Thurman (2018), CEs are the dominant US mechanism for private land conservation, and “the government’s main role is to set tax policy and then let individual landowners and nonprofit organizations determine the quantity and patterns of permanent conservation under limited regulation” (p. 338). While there is seeming separation of CEs and governmental mandate (local, state, or federal), tax-setting and ensuing property taxation can create use pressure or conversion, as “many public policies influence private land conservation both in the United States and elsewhere… [including] the various incentives built into tax codes that differentially tax developed and undeveloped land” (Parker & Thurman, 2018, p. 338). The tax appraisal’s land use designations (LUDs) for CE properties are malleable social constructs that are guided by factors beyond the land’s innate physical characteristics. These include a combination of governmental decision-making through aspirational land use planning and policies; social and ethical will; adjacent land uses; market forces; and economic valuation of current use. There is an inherent bias in property taxation toward the highest and best use of a property manifested in a governmental assessor’s LUD (CA State Board of Equalization, 2002, ch. 5). Governmental property tax appraisal takes several forms (i.e., market, cost, or income approaches) (CA State Board of Equalization, 2002, ch. 6), but generally follows the definition of highest and best use from The Appraisal of Real Estate, as: “the reasonably probable and legal use of vacant land or an improved property, which is physically possible, appropriately supported, financially feasible, and that results in the highest value” (Lennhoff & Parli, 2020, p. 198). This is conceptually economic and anthropocentric, focused on a single type of productive and legally allowed use by which the property has been designated through governmental land use controls. However, the LUD for property taxation is not the same as the zoning district in which the property exists; the zoning determines the possible types of land uses, and the appraiser considers the actual highest and best use within the constraints of the property’s zoning.

The CE parcel(s)’ LUD(s) may not be a topic that LTs traditionally consider, but the LUD *can be* a motivator for the property owner to use or convert the land to a higher intensity use or can influence the CE placement decision to alleviate a tax burden. Consequently, the LUD is a way in which the CE and the parcel’s land use can support conservation objectives, especially where they are coincident—and sometimes where they are not (Brunson & Huntsinger, 2008). The LUD may be a proxy for the property’s use—whether actual or avoided—and is an unacknowledged but powerful land use mechanism with the potential to affect the CE in ways that warrant further exploration.

Unlike classification systems for public protected areas that describe their allowable land uses and varying degrees of dedication to biodiversity protection (IUCN, 2004), there is little empirical understanding about the prevalence of different forms of protection from CEs. This is because CE parcel(s)’ LUDs often belie prescribed classification and there is inherent flexibility and individual onus that can be drafted into the CE instrument. Some sources of variability include the heterogeneity of CE purposes; the LT standards for and tolerance of existing and future land use intensity on CE parcels (Cross et al., 2011); the tax assessor’s LUDs for CE parcels and potential to manifest that use, depending on societal and political will; a range of biological impacts associated with varying degrees of private discretion in land ownership decisions and use(s) across the array of diverse CEs instruments; and an inherent property tax bias that creates an incentive for some landowners and easement holders to put the property to economic and anthropocentric (e.g., single-family development, natural resource extraction or cultivation, etc.) use (Brunson & Huntsinger, 2008).

The CE instrument itself is purposively heterogeneous, reflecting a breadth of permissible and socially desired reasons under US regulations and state CE enabling acts that include “agricultural, forest, recreational, or open-space use, protecting natural resources, maintaining or enhancing air or water quality, or preserving the historical, architectural, archaeological, or cultural aspects of real property” (UCEA, 2007, Section 1 (1); McLaughlin, 2017). The intentional CE purpose diversity increases the instrument’s versatility and ubiquity on a myriad of private lands with a range of LUDs. The CE may support the purposes of the LUD, or they may respond to the LUD, challenging its effect.

Many CEs were not intended for biodiversity protection; of the 119 CEs held by the Nature Conservancy in eight US states, almost half were working landscape easements and allowed anthropocentric uses (Rissman et al., 2007). Brunson and Huntsinger (2008) and Morris and Rissman (2009) affirm the Rissman et al. (2007) findings in additional studies in other locales. Some land uses in private ownership are more compatible than others in realizing mutually complementary objectives, especially since urbanization and agricultural uses can degrade the natural habitat and change the native species composition (IPCC, 2019; Lindenmayer, 2019; Reiner & Craig, 2011). They can also effectuate biodiversity protection, depending on the level of development density and the intensity and spatial placement of use (Farr et al., 2017). Ranching and some CEs’ conservation objectives may be compatible, with a co-occurrence of interests such as habitat and open space preservation, grasslands maintenance, ecosystem services, invasive species removal, and so forth (Braza, 2017; Brunson & Huntsinger, 2008; Reiner & Craig, 2011; Rissman & Merelender, 2008).

Concomitantly, there are no uniform standards in the United States for the land uses on the properties eligible for CE placement. Instead, they are subject to criteria (if any) established by the LTs and other qualified CE holders at the time that they agree to hold the CEs. The Land Trust Alliance (LTA) has a rigorous LT accreditation program that creates accountability via LTs’ standards for CE acceptance, but accreditation is voluntary and each LT maintains mission-based autonomy. The CE property’s existing land uses and the ability to continue or intensify them may also have important for biological protection. In 269 CEs from six US states, those that were granted after 2000 showed an increase in intensive land uses (building of structures, bounded timber harvests, and grazing) or were drafted into the CE grantor’s permitted uses, reserving the ability to increase the uses over time (Owley & Rissman, 2016).

Unlike public protected areas with overt designations and associated management objectives, it has been challenging to make broad statements about CEs, the LUDs on their parcels, and their biological impacts given the volume of factors that influence where, why, and how CEs manifest in the privately-owned landscape. Better understanding of the CE manifestations and the variability in its forms of flexibility could help to establish a classification system with permitted LUDs and levels of biological protection on CE parcels. Focusing on one facet of that CE flexibility, our study goals were to (1) discern whether there are patterns in the tax assessors’ LUDs of CE parcels, which might motivate CE placement or impact their biological protection potential and/or purposes; and (2) determine whether these trends could contribute to an emerging classification system like that for public protected areas. We systematically examined the trends (but not causality) in the county tax assessor LUDs of CE parcels to (1) identify categorical prevalence (if any) associated with CE use in 12 exurban areas; and (2) evaluate the extent to which CE purposes and a CE parcel’s LUD for tax appraisal are correspondent or divergent.

## 2 | METHODS

To meet our study goals, we created fine-scale CE datasets for 12 counties in six US states (CA, CO, MN, PA, SC, and VA). These include Sacramento and Sonoma in California, Boulder and Mesa in Colorado, Douglas and Washington in Minnesota, Lebanon and York in Pennsylvania, Charleston and Greenville in South Carolina, and Albemarle and Loudoun in Virginia. Within each county, we isolated the parcels on which the CEs were located for the period of study (between 1997 and 2008 or 2009, depending on data availability). We defined these parcels as those with a CE that overlapped or covered any part of that parcel. There are often one-to-one parcel-to-CE relationships, but a CE may also cover with multiple parcels or only part of a parcel. We re-coded the county-level tax appraisal LUDs with a master LUD guide to create uniformity and categorical breadth across counties, as each county assessor has its own variations on types of land uses, level of appraisal detail, and corresponding zoning ordinances (Appendix S1). We define “LUDs” as the land uses for which the property is being assessed/taxed (its highest and best use within the constraints of the county or city zoning) by county recorder’s offices/local tax assessors and modified for comparative uniformity across the counties.

To briefly summarize our evaluation approach for these Granular Conservation Easement Datasets (GCED) for 1997–2008/2009, we ran our analyses on the master LUD and the size of each CE parcel both within and across the combined counties. We validated these analyses by checking the proportion of the LUD categories for the CE parcels against the dominant LUDs both across the counties and within the counties over time, using a Bayesian generalized linear mixed model. The analysis and dataset construction details are presented in the following subsections.

### 2.1 | GCED county selection and construction

We used the following three primary selection criteria/guiding principles for state and county choice, which were designed to meet multiple research objectives in a larger project. First, our counties needed to be within each Census region of the United States. and within the states with the highest numbers of acres preserved in CEs in each, without normalizing by urbanized area. They needed to exhibit the following: urbanization pressures (i.e., growth), CE quantity by county (the greater the better, but at least more than 25 for statistical viability), and an exurban character. We used the “exurban” definition as a guiding but not entirely definitive principle (i.e., greater than 0.15 people/ha (0.06 people/acre) or “exurban” density as 0.68–16.18 ha per unit) (Theobald, 2001). Accordingly, we captured residential land use beyond the urban–suburban fringe that comprises parcels/lots generally considered too small to be productive agricultural land use.

Our second selection criterion was Level 1 ecoregion exemplification, choosing the biggest and most representative ecoregions in the contiguous United States, including Marine West Coast Forest, Western Forested Mountains, Mediterranean California, North American Deserts, Great Plains, Eastern Temperate Forests, and Tropical and Subtropical Coniferous Forest. US ecoregions are spatially hierarchical; Level 1 is the largest and conceptually closest to biome (Bailey, 1995). Our third criterion was to maintain a balance of states with state easement enabling laws that mandate some form of land use planning principles/public oversight in CE placement. We coded and completed the datasets for the counties, using manual coding with intercoder reliability measures for 2823 CE documents, including the CE reasons.

The GCED consists of all available CEs placed during (and preceding, if there is an amendment) 1997–2008/2009 in the county recorder’s offices, with the caveat that it was difficult to locate those that were more broadly categorized as a “deed” rather than a CE.

### 2.2 | Master LUD process

For the master LUD process, we sought to preserve detail while creating generalizability. The counties’ assessors each have their own set of LUD categories linked with their taxation structure and state legislation. To compare across counties, we coded each county with an LUD built on a general appraisal value category (i.e., 1000 for residential), and then additional sub-categories for specific LUDs (i.e., 1100 for single-family residential 1–4 units, 1101 for single-family dwelling within residential and single family residential 1–4 units, etc.) (Appendix S1

dix S1).

Critically, there are no assessor land use categories specifically for open space or biological protection on private land in our counties of study; even where the land is forested, it is still assessed as a source of timber. There are public government categories for wildlife parks and recreation (state and county) and greenbelts, and there are categories that generally relate to preservation on private land, including agricultural preserve and timber preserve, and conservation and conservancy (Appendix S1). But there is no distinct category identifying economic value from biological protection since ecosystem services often lack a traditionally defined market. Instead, the governmental appraisal guidance recognizes open space restrictions for agricultural lands, wildlife habitat, and parks, and utilizes preferential assessment to reduce/limit valuation accordingly. But the approach is state-dependent (e.g., CA State Board of Equalization, 2002, p. 16), and these lands are not entirely exempt, unlike some other societally valued uses (e.g., churches and schools). Additionally, vacant land is treated as the potential highest and best use under the appraisal definition and is taxed accordingly.

### 2.3 | Limitations in the GCED construction

We have the basic information associated with each parcel (size, value by year, LUD, etc.) but lack the ownership information as part of our anonymity agreements with the counties. Consequently, there could be multiple small parcels held by a single owner with a conservation management strategy across them, despite their LUDs, and it would not be revealed in our analysis. Parcels with CEs during the period of analysis may have been omitted because of recording problems in the recorder’s offices (Morris & Rissman, 2009). Accordingly, we triangulated information with other existing datasets (i.e., the National Conservation Easement Database), the spatial layer of CEs, and information from LT websites. The parcels also changed over time, with some dropping out—whether from lack of taxation/change to an exempt category or because they were subdivided—while others are created during the period for the same reasons. Through our CE spatial layer and associated parcel cleaning, we eliminated parcel duplication by verification with legal descriptions (metes and bounds, township and range), and manual curation of over 6322 records.

Some parcels did not have an LUD prior to the CE placement, which may be attributed to parcel subdivision and concurrent CE placement. To determine whether the subgroup without a master LUD prior to CE placement was like those with both a pre- and post-LUD, we compared the proportion of parcels in each LUD category in the post-CE using chi-square tests. Our results showed sporadic difference by year and by county, without a consistent trend or pattern across them. Consequently, we ran analysis on each county for the summary statistics noted below, and across the combined counties, with this acknowledged bias. We also conducted more refined assessment of the pre- and post-CE LUD subgroups, as noted below.

### 2.4 | Poisson regression evaluating LUD count representativeness

To assess the representativeness of the underlying land use counts within and across counties, we used a Bayesian generalized linear mixed model that was fit using a Markov chain Monte Carlo sampling algorithm. The model is constructed as follows. Let $Y_{st}$ denote the number of agricultural (residential) CEs in the sth county at the tth time point and let $n_{st}$ denote the corresponding total number of CEs, for S=1,…,S. To investigate both population and county-level trends, we assumed that:

$$Y_{\text{st}}∼\text{Poisson}\left\{n_{st}\exp\left(\beta_{0}+\beta_{1}t+b_{0s}+b_{1s}t\right)\right\},$$

where exp(⋅) denotes the usual exponential function, $\beta_{0}$ and $\beta_{1}$ are population level regression coefficients, and t denotes the time at which $Y_{st}$ was observed. Here $b_{0s}$ and $b_{1s}$ are random effects allowing for a shift away from both the population level intercept and slope for each county. We assumed that $b_{js}∼N\left(0,\sigma_{j}^{2}\right)$, for j=1,2 and S=1,…,S, and that $b_{js}$ is independent of $b_{j^{\prime }s^{\prime }}$for all $j\neq j^{\prime }$ and $s\neq s^{\prime }$. To complete the model, we specified the following priors: $\sigma_{j}^{-2}∼Gamma(1,1)$, $\beta_{0}∼N(0,2)$ , and $\beta_{1}∼N(0,2)$. Based on these specifications, we constructed a Markov chain Monte Carlo sampling algorithm to draw a sample consisting of 10,000 realizations from the posterior distribution. We used this posterior sample to estimate and draw inference on the population-level trend $\left(\beta_{1}\right)$ as well as the county-level trends $\left(\beta_{1}+b_{1s}\right)$ in the usual manner.

### 2.5 | Analysis approach for trends in CE parcel LUD and CE purpose

We calculated the following statistics for the nominal master LUD and continuous parcel size variables, by year, to meet our study goals: summary statistics (mean, median, mode, standard deviation, variance, skewness, and range), measures of dispersion (for nominal variables, frequency distribution, percentages, IQV; for continuous variables, range, variance, standard deviation, percentiles, interquartile range), and measures of variability (coefficient of variance, standard error). We assumed that vacant but designated should be treated as the designated use because the appraisal literature does the same (CA State Board of Equalization, 2002). This means that vacant agricultural is treated as agricultural, while vacant residential is treated as residential. We recognize that there can be ranges in intensity associated with the kinds of residential and/or agriculture that are eligible for placement and their surrounding uses. Additionally, CEs can be present in an area zoned and appraised for a particular use, but that use is restricted in the CE language, and the LUD will remain the same. To determine if mean CE parcel size differs significantly by county, we performed a one-way ANOVA on the CE parcel size (Appendix S2).

There are several ways to examine data with both CE and parcel unique identifiers. Accordingly, we conducted the descriptive analysis three times; first, by choosing the parcels that had a CE at some point during our time period, revealing the basic LUD trends on these parcels. Most of our analyses focused on this approach. Second, we chose the parcels based on the unique CE identifiers (meaning that a parcel could have more than one CE, creating repetition in the parcel counts), revealing the parcel LUDs associated with a CE each year. Third, to conduct a more refined assessment of the pre- and post-CE LUD sub-categories by differentiating the pre- and post-CE placement analysis, we created subsets of those with pre- and post-CE LUDs in each county for comparison, and we created subsets of those with no pre-LUD in each county. We ran the same statistics noted above on these subgroups, as well as Fisher’s Exact test to examine the relationship between the pre- and post-CE placement LUDs.

To determine whether there was a relationship between the CE purpose and the CE parcel LUD, we ran a chi-square test (*p* <.05) on the first listed CE reason and the first listed master LUD for the year in which the CE was added to the parcel. We ran these tests on all CEs in each year in each county and across the combined counties. We acknowledge the embedded assumption that the order of listing implies primacy in using both the first CE reason and the first listed LUD. We note that a significant association does not necessarily indicate *similarity* of CE purpose and LUD; instead, it indicates the presence of a pattern between the MLUDs and the CE reasons by county.

## 3 | RESULTS

### 3.1 | LUD trends across all counties

**FIGURE 1:**
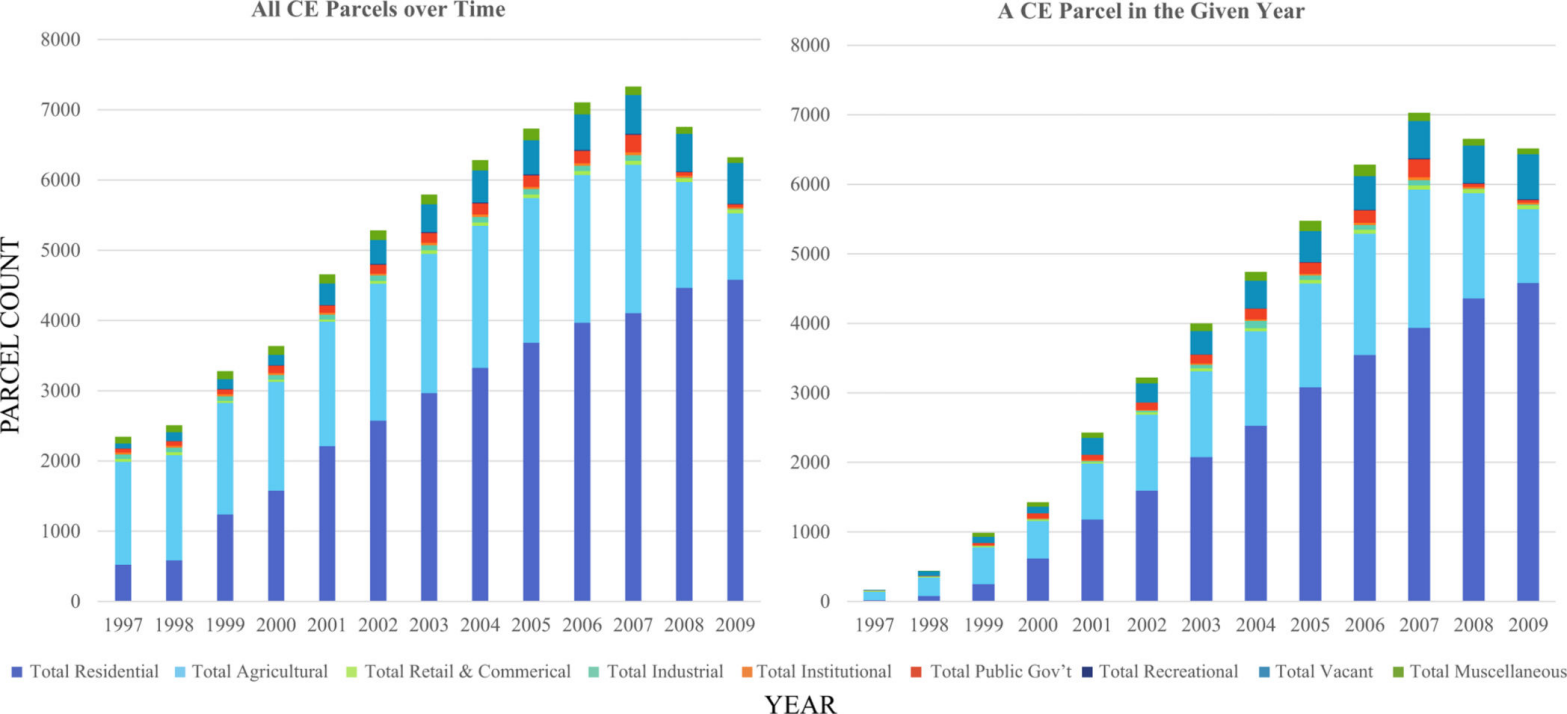
Consolidated trends in the LUDs over time for the CE parcels across the combined counties.

Our results identify that most LUDs associated with CE parcels are residential and agricultural. There is a distinctive trend across the counties over time that demonstrates an increase in CE parcels with residential LUDs (Figure 1 and Appendix S3). Across the counties, the CE parcels’ top two LUDs with counts of 50 or greater in any given year are residential (single-family dwelling, vacant residential), followed by agriculture (Appendix S4). To starkly illustrate this trend, we consolidated the LUDs into their most fundamental grouping (residential, agricultural, etc.), showing that residential is the dominant LUD (Figure 1). It surpassed the agricultural parcel count in year 2000, whether for all CE parcels over time or parcels with a CE each year.

The top three LUDs on parcels with a CE across all counties in our timeframe were in agricultural and residential uses. Agricultural uses are highest in early years (1997–1998), after which the dominance switches to residential in 1999–2009 (Appendix S4). The LUDs for parcels with a CE each year show a slightly more abrupt and extreme shift from agricultural to residential dominance by sheer parcel count, and it occurs in 2000, rather than 1999 (Appendix S4).

In the Poisson regression analysis, the population-level trend parameter$\left(\beta_{1}\right)$, which represents a global rate of change, was not significant for the residential or the agricultural CE parcels (Table 1). This suggests that there are no global time trends in the increase/decrease in CE parcels (Table 1). Although not shown in the Table 1 results, additional Poisson regression found that only the public government and institutional properties and unknown LUDs had significant time trends across the combined counties as a group; the unknown designation decreases over time, with LUDs being assigned through subdivision and parcelization.

### 3.2 | LUD trends by county

**FIGURE 2:**
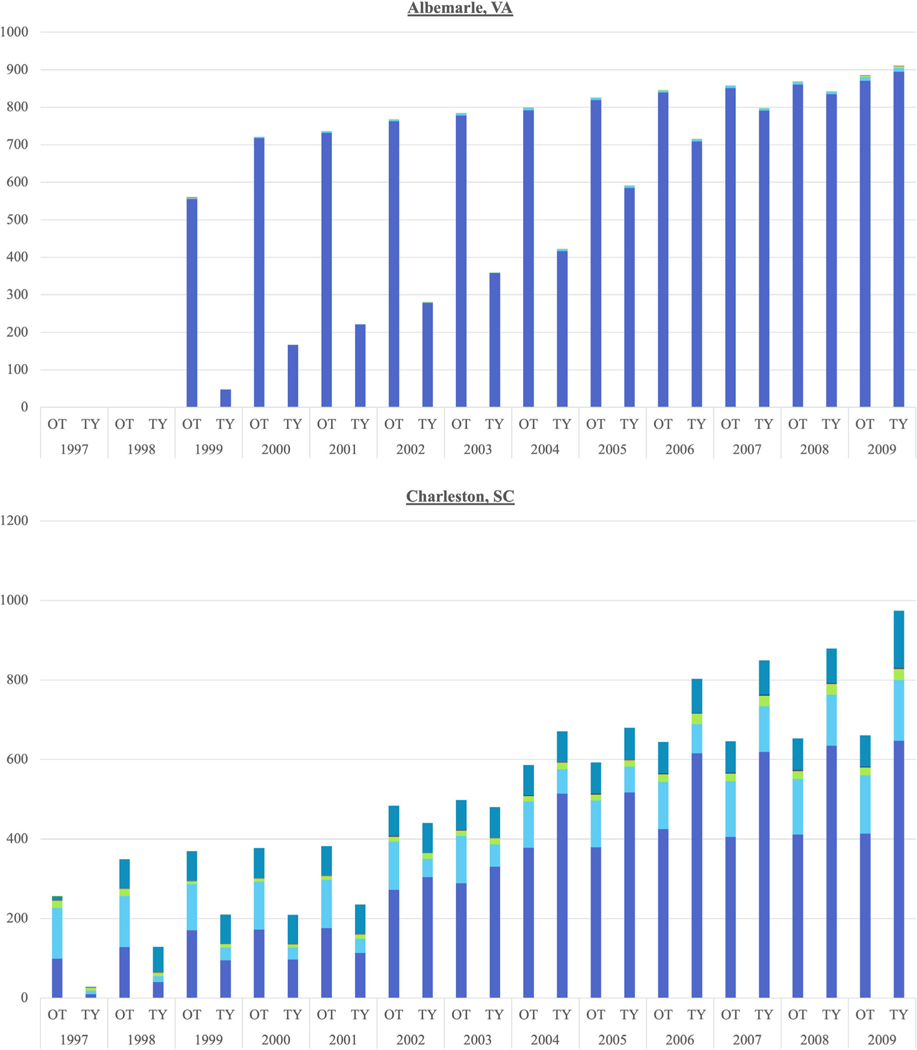

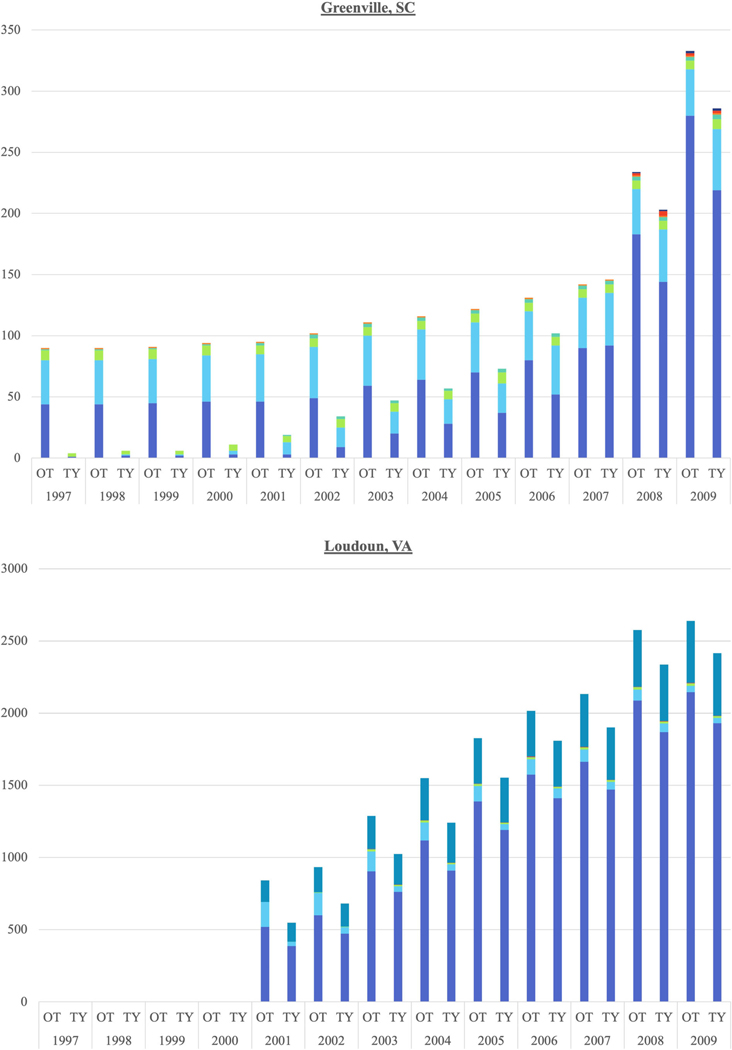

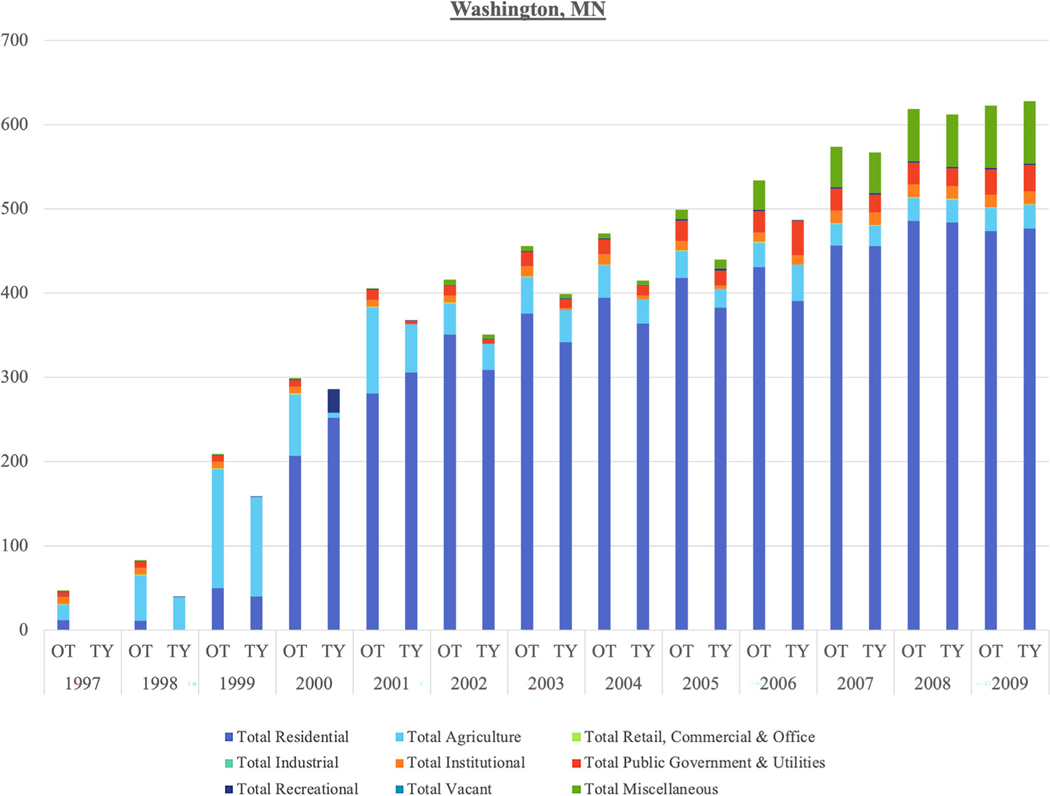
Counties with residential LUD dominance over time, by parcel. OT = LUDs for all CEs over time. TY = LUDs for parcels with a CE in that year.

**FIGURE 3:**
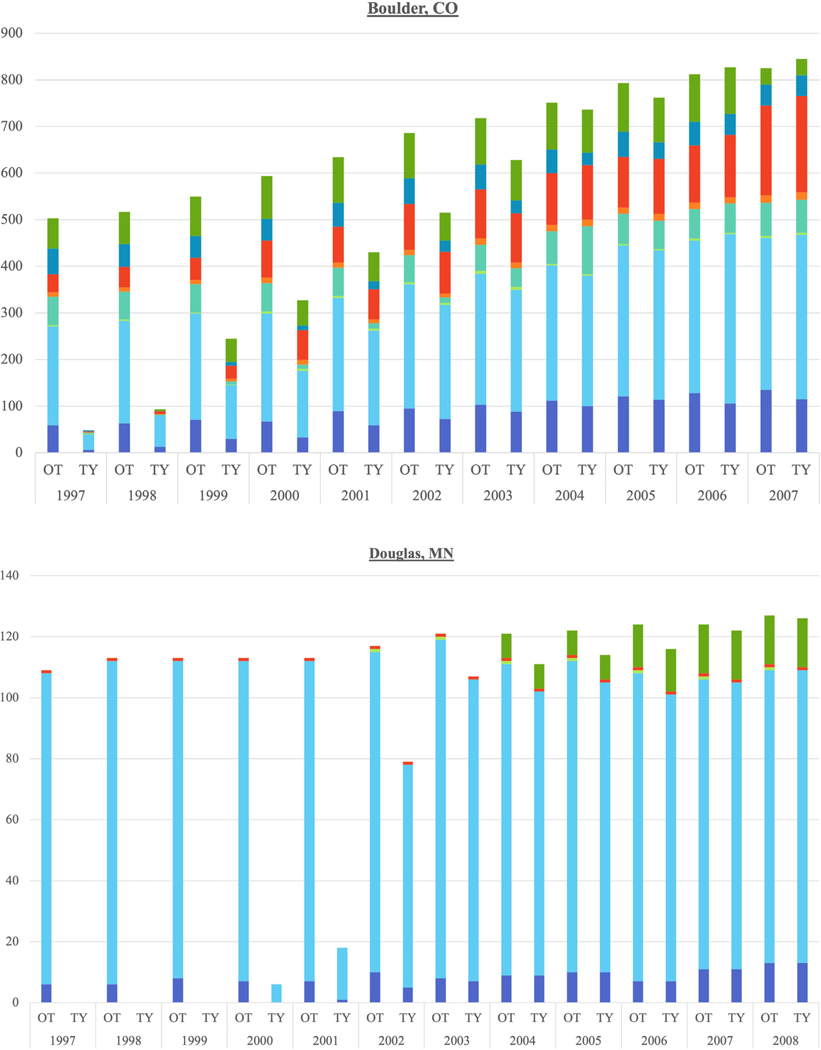

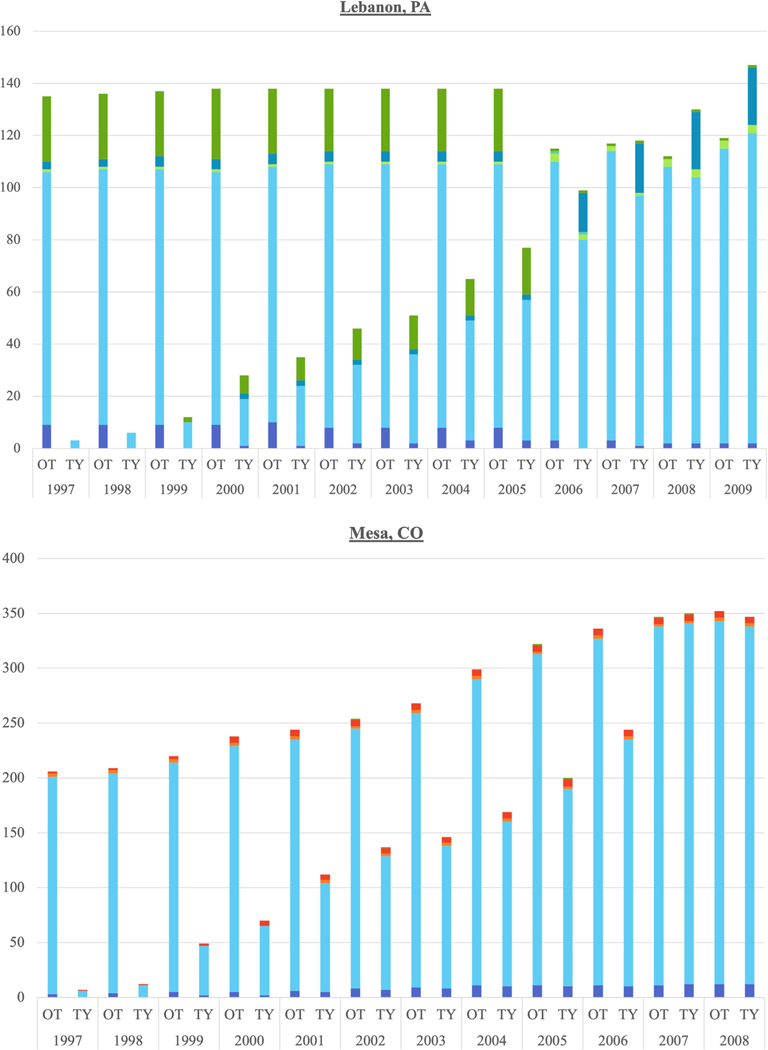

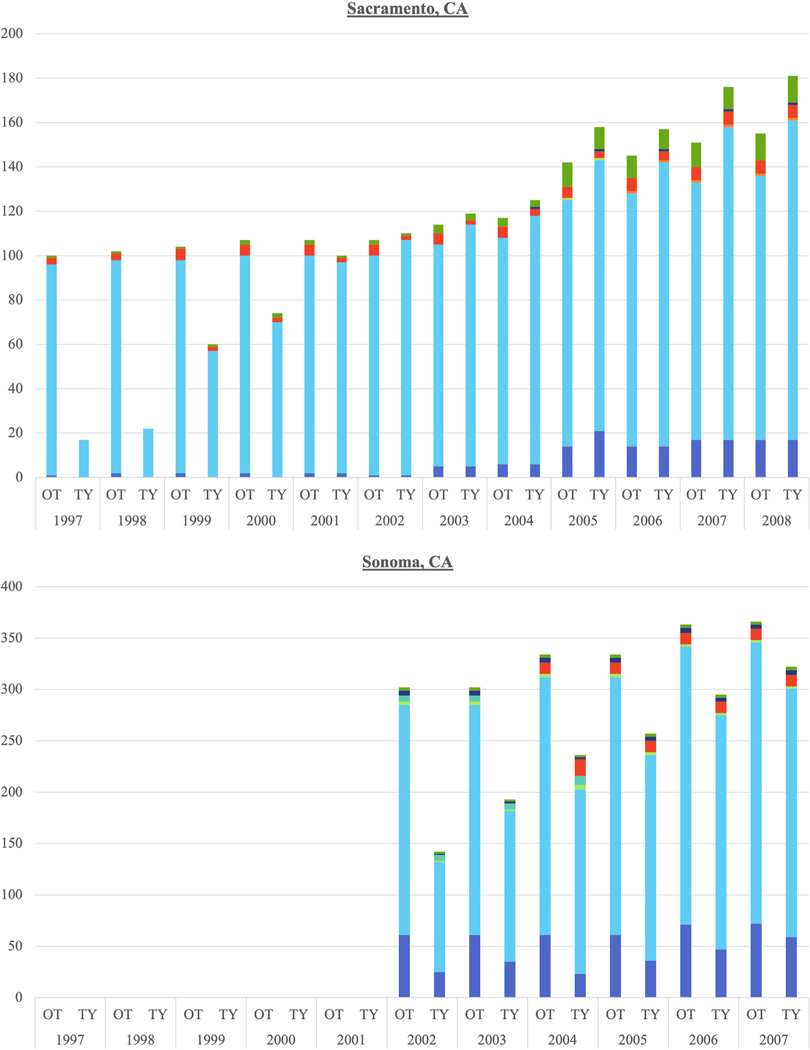

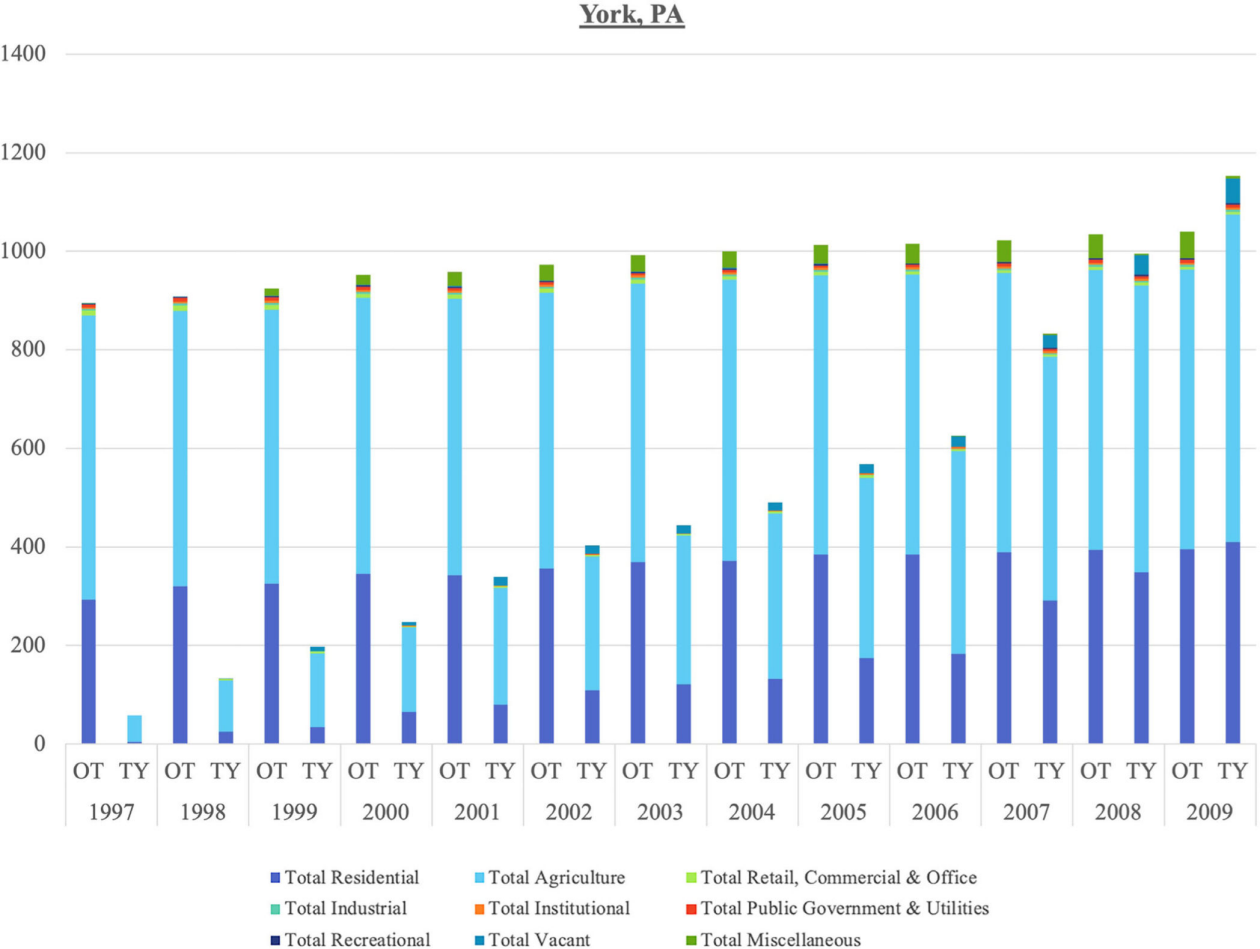
Counties with agricultural LUD dominance over time, by parcel.

When further parsing the land use change trends in each county separately, the top three LUDs divide the counties into two groups over the 1997–2008/2009 time period: those with dominant residential (Albemarle, VA; Charleston, SC; Greenville, SC; Loudoun, VA; and Washington, MN) (Figure 2) and those with dominant agricultural uses, despite a residential creep (Boulder, CO; Douglas, MN; Lebanon, PA; Mesa, CO; Sacramento, CA; Sonoma, CA; and York, PA) (Appendix S4 and Figure 3). Although they do not perfectly correspond to a geographic region or ecoregion, the residential dominance is present in South-eastern states (VA and SC) while the agricultural dominance is in the West (CA), Mountain (CO), and Northeast (PA) (Figures 2 and 3).

The Poisson regression within each county shows that Albemarle, Boulder, Charleston, Greenville, Lebanon, Loudon, Sacramento, Washington, and York had residential counts that were significantly different than the general increase in each LUD for the general population of CE parcels over time (Table 1). All of those, save Lebanon, are significantly increasing. This significance matches the dominant residential trend in some of the counties, but three agriculturally dominant counties, Boulder, Sacramento, and York, also experienced increasing time trends in the number of residential CE parcels, affirming the larger preponderance of residentially designated CE parcels across the combined counties (Table 1 and Figure 1). Although Lebanon was also agriculturally dominant, it experienced a significantly decreasing time trend in residential CE parcel count.

Within each county, Charleston was the only county with coincidence of significantly increasing agriculture over time and significant residential counts. Three of the counties with dominant residential land uses over time and positively significant residential time trends had significantly decreasing agricultural time trends (i.e., Greenville, Loudoun, and Washington). York was the only agriculturally dominant county to show a significant decreasing time trend in agricultural CE parcels. In contrast, two agriculturally dominant counties, Mesa and Sonoma, showed significantly increasing agricultural time trends (Table 1).

### 3.3 | LUD change after CE placement

We refined this analysis by examining the subset groups of CE parcel LUDs pre- and post-CE placement, and the CE parcels with a LUD-only post-CE placement. This revealed the composition of the dominant LUDs, both pre-and post-CE placement; whether the original LUD shifted once the CE was placed; and whether there was any difference (by dominant land use category, size of parcels, etc.) between the subset groups of pre- and post-CE, and post-only. In the pre- and post-LUD subset, agriculture was the dominant LUD by parcel count prior to CE placement in nine of the 12 counties (Table 2). And it remained the largest category post-CE placement in six of the 12 counties. Douglas, Mesa, Sacramento, and Sonoma are the same counties that showed agricultural dominance in the overall CE parcel count; post-CE placement, Boulder shifted to public government and utilities/county. Both Loudoun and Washington were agriculturally dominant prior to CE placement, but Loudoun switched to vacant/miscellaneous vacant and Washington became dominant residential after CE-placement. The other dominant pre-CE LUD is residential in three of the 12 counties (Albemarle, Charleston, and Greenville) and that trend remained after CE placement.

Only two counties showed a high percentage of parcels with LUD change after CE placement (Loudoun (67.66%) and Washington (64.95%)) (Table 2). Approximately a quarter of pre- and post-LUDs changed in five other counties (Boulder (31.97%), Charleston (23.02%), Douglas (25%), Greenville (29.59%), and Lebanon (27.13%)), yet their top three LUD categories remained the same—except in Boulder. The designations in the other five counties (four agriculturally dominant and one residentially dominant) remained virtually the same, with less than 7% change post-CE placement.

Comparing the LUDs for the two parcel subset groups in each county showed that the majority (10 counties) have the same post-CE dominant land use category (Table 3). Only Boulder and Loudoun displayed a difference between the subsets, with agricultural dominance in the Boulder post-only subset, and residential dominance in the Loudoun post-only subset. Additionally, the post-only LUDs are solely agricultural (six counties) and residential (five counties); Douglas had no post-only data, so it is omitted from the table. The mean size of the post-CE-only parcels is smaller than those in the pre- and post-CE subset group in six of the counties; Lebanon, Mesa, Sonoma, Washington, and York have slightly larger mean parcel sizes in the post-only group. Of those, Washington is the only residentially dominant county, while the rest are agriculturally dominant. This differentiation between parcels with a pre- and post-LUD and those with only a post-LUD generally mirrors the trends in the combined analysis.

### 3.4 | CE parcel sizes in aggregate and by LUD

The mean size for parcels with a CE in a given year are almost all smaller than the recommended minimum open space area (150 ha) for conservation developments (CD) to support biodiversity conservation, except in Greenville (Table 4 and Appendix S5) (Farr et al., 2017). It is important to note that a CD is comprised of multiple parcels, and the residential parcels may be significantly smaller than the conserved open space area(s). Or there may be smaller residential parcels with a percentage of conserved space that in total, achieve the 150 ha, rather than relying on a single conservation parcel. Nonetheless, Greenville, with residentially dominant LUDs, had the largest mean parcel size in any given year (196.38 ha in 2003), followed by Mesa, Sacramento, and Sonoma, which are predominately agricultural.

The medians suggest that mean CE parcel size is skewed by a few larger parcel outliers, especially in some counties (Appendix S6). Within county, the median parcel size falls into one of three trends. These are the residentially dominant LUD counties, and they have medians that are consistently lower than 8.09 ha (20 acres) in all years, including Charleston (appreciably lower in each year), Greenville (particularly when compared to its mean, which is the highest of the counties), Loudoun, and Washington. The second trend is medians that are consistently higher than 8.09 in all years, including Albemarle, Douglas, Lebanon, Mesa, Sacramento, Sonoma, and York. All but Albemarle are agriculturally dominant in LUD count. The third trend is a median that starts out higher than 8.09 but drops below over time; Boulder is the only county to follow this pattern, with median values below 8.09 ha from 2002 to 2009, despite being agriculturally dominant. Across the counties, the medians are consistently lower than 8.09 ha each year, ranging from 0.26 at its lowest in 1997 to 3.77 at its highest in 2008 and 2009 (Table 4 and Appendix S6). These data show that despite the higher count in residential LUDs, the agriculturally designated parcels are larger across the counties (Appendices S4 and S6).

When comparing mean parcel size by LUD, although lower in parcel count, recreational land use had the highest mean parcel size of the LUDs at greater than 70 ha (Appendix S6). This affirms Brenner et al.’s (2013) findings, although agricultural LUDs, the second highest in parcel count, had a mean slightly lower than 70 ha. Residential was the third lowest in mean size but highest in parcel count. These results are qualified by the fact that we have not empirically tested the difference between the parcel sizes in the remainder of the counties—in part, because the parcel sizes within the urbanized areas are likely to be smaller. There is definite variation across and within the counties, illustrated by the standard deviation, and by one-way ANOVA of the means of the parcels with a CE at some point in time during our timeframe (Appendix S2).

### 3.5 | CE parcel land use and CE purpose compatibility

The counts in the first listed CE reason over time (1997–2009) across the counties show open space preservation as the highest (20.30%), followed closely by agricultural viability/livestock (18.53%), and then scenic value, natural condition, and governmental conservation policy/open space program under the federal tax code for CE purposes in smaller volume (9.24%, 7.14%, and 6.79%, respectively) (Appendix S7 and Table 4 ). The other reasons are depicted on other graphs within Appendix S7.

When examined over time within the counties or across the combined counties, the first listed CE purpose and its parcel LUD do not have consistent significant associations with each other (Table 4 ). Only six of the 12 counties show a significant association between the first listed CE purpose and the LUD. Of those, three have predominant residential LUDs and the CE purpose is disparate (i.e., open space preservation, natural condition/resource values, or scenic value/beauty) (Loudoun, Greenville, and Washington). The other three have predominant agricultural LUDs and the same CE purpose (i.e., agricultural viability/livestock) (York, Boulder, and Mesa). Analysis over time across the combined counties shows a significant association between the predominant LUD (agriculture from 1997 to 1998, and residential from 1999 to 2000) and the first CE purpose (open space preservation). However there are six counties without a significant relationship and their first CE reasons vary (Table 4 ). When regionally grouped, there is little consistent pattern of significance, except that two agriculturally-dominant counties with a significant association are in the same state (CO). Also in the western region of the United States, two counties in the same state (CA) do not have significant associations. But the rest of the counties differ from the other county in their state. Additionally, the first listed CE reason by highest percentage in half of the counties is agriculturally related (either agricultural viability/livestock, or soil), all of which also have agriculturally dominant LUDs. Three counties have open space preservation or natural conditions as their dominant purpose, and two of the three are residentially dominant in LUD. Only two counties (Albemarle and Charleston, both residentially dominant) have a majority of biologically related first CE reasons (Table 4).

## 4 | DISCUSSION

We have established a baseline of information about and revealed patterns in tax appraisal LUDs of CE parcels over a decade (1997–2009) in geographically- and eco-regionally-representative exurban areas with growth pressure. Our results show that the predominant LUDs assigned through local policy (i.e., the assessor’s office) are highest by count in residential—whether a single-family dwelling, vacant but designated residential, or residential generally, followed by agricultural working lands (Levin, 2014) (Appendix S4). There are also distinct sub-trends when assessing the counties individually, with the top three LUDs in two groupings over the 1997–2008/2009 period: those with dominant residential and those with dominant agricultural uses. The agricultural properties, while lower in overall count, are larger in physical size than the residential parcels across the counties over time. Recreational parcels were so low in annual count (10 or fewer per year) that they barely manifested in Figure 1, but they are physically the largest of all the CE parcel LUDs. In terms of the assessor-assigned LUD and its relation to CE placement, CEs are employed on agricultural aka “working” lands, and on residential properties, both in the cross-county and within-county examinations (i.e., pre- and post-CE placement LUDs and post-only LUDs).

It is integral to understand these LUDs for CEs because counties make the designation based on current or potential economic parcel valuation and tax accordingly, possibly incentivizing land conversion. While manifestation of a particular land use is not inevitable, the tax category can induce use even after CE placement, unless expressly prohibited in the CE (Brandt, 2014; Polyakov & Zhang, 2008). The CE has the power to complement an LUD (e.g., a CE with agricultural purposes on a parcel with an agricultural LUD) or to frustrate it (e.g., a CE that prevents residential subdivision on a parcel with a residential LUD), depending on both the LT and the property owner’s intent (Parker & Thurman, 2018). Some economically valuable land uses are compatible with biological protection objectives (Farr et al., 2017), but some are overtly detrimental. The IPCC (2019) report on land finds that current conversion to residential and agricultural land uses contributes to biodiversity loss, but it is not clear whether CE placement in the GCED is a response to the existing LUDs to improve/restore biodiversity, or for another reason entirely (e.g., counteracting development pressure on agricultural lands). Other findings suggest that preferential assessment to minimize appraisal utilization only slows land conversion on non-CE lands (Brandt, 2014; Polyakov & Zhang, 2008), while CEs may prevent it.

Instead, there is an argument for tax appraisers to institute a biological conservation exempt designation for private lands that resembles the approach to other societally valued services (e.g., churches, governmental institutions, etc.). This would acknowledge the broader societal value from climate change and biodiversity protection that manifests in such a land use. Parker and Thurman (2018) identify the conservation versus development value issue as “an incentive misalignment… because easements are appraised by foregone development values rather than by the value of the public-good amenity flows generated from undeveloped land” (p. 345). It is a novel approach to effectuate externality internalization that is distinguished from assessing a land area tax instead of a land value tax (Brandt, 2014). As Brandt (2014) states, “there is a long tradition of granting property tax exemptions to people or activities that are viewed as vulnerable or to promote desirable activities. Educational, religious and charitable institutions are exempted from property taxes in many countries” (p. 14). This designation could correspond to CE placement; depending on the biological value of the ecosystem services from the unconverted land use, a property owner might receive payment in addition to the tax exemption. This would further solidify a market-based approach to valuing ecosystem services. Already, two of the counties show a change toward an LUD with lower appraised value post-CE placement (Boulder and Loudoun), suggesting potential for compensatory policy actions such as subsidized conservation land uses. However, there are substantial caveats to instituting additional fiscal subsidies, including significant considerations of CEs’ physical location in connection to other conserved lands (connectivity, habitat conservation potential) and the social value of their relative public access. Based on this and other research, optimal policy approaches would weigh individual fiscal benefit for the CE landowners, social detriment from permanent displacement of other societally valued land uses, and social value from potential ecosystem services (Merenlender et al., 2004).

Nonetheless, the GCED are poised to address the issue of valuation pressure driving land use because the study locations are exurban areas with competing societally valued land uses. Our county selection rationale was based on the rapid and spreading pattern of exurban growth and the use of CEs to preserve the remaining areas of social and ecological value as defined by the LTs and the federal tax code (Theobald, 2004). Our county selection criteria included evidence of a positive growth rate, which could impact some of the residential significance within the counties. Regardless of county or state, there is an evident trend in the tax appraisal-based LUDs associated with 2823 CEs. However, we are unable to state whether there is compatibility between the LUDs and the conservation objectives in the CEs since only six counties showed significant association. In those, the corresponding LUD was agricultural, with an agricultural CE purpose for three counties. Where the residentially dominant LUD counties had significant association with their CE reasons, those reasons included: open space preservation, natural condition/natural environment/nature resources values, and scenic value/beauty. These patterns suggest an association between working lands (i.e., agricultural LUDs and agricultural CEs) but more variability in the kinds of CE reasons and residential LUDs.

Consequently, the LUD should be considered a basic element if creating a classification system for private land conservation strategies, particularly with CEs that continue to permit anthropocentric uses. The intensity of use that results from tax appraisal, coupled with parcel size and CE purpose, can impact biodiversity protection objectives in several ways. The sheer volume of smaller residential parcels suggests that the type of residential development in the counties may not conform to lower environmental impact conservation subdivision designs (Farr et al., 2017). The variability in the significance of the association between the tax assessor’s LUD and the first CE reason for individual counties suggests that there is no definitive trend in their compatibility. Even if remnant areas have ecological value (Wintle et al., 2019), there is a well-established literature on biodiversity outcomes from larger versus smaller habitat areas and the benefits of reduction in human-species interactions (Laurance, 2009; MacArthur & Wilson, 1967; Soulé & Simberloff, 1986).

More importantly, the predominant and primary CE reason over time is open space preservation, which may promote—but is not overtly intended for—biodiversity/habitat protection. This finding challenges an implicit assumption that CEs promote ecological conservation, biodiversity, and habitat preservation (Bastian et al., 2016; Kiesecker et al., 2007; Stroman & Kreuter, 2014). However, open space preservation is the second reason cited for CE use in Kiesecker et al.’s (2007) study and, as Hilty et al. (2006) and the IPCC (2019) note, it may be increasingly imperative in a time of land use fragmentation/change and climate change. Additionally, a CE’s primary purpose does not preclude complementary purposes (which were not assessed in this article), and most CEs in the population had numerous listed purposes. Open space preservation is compatible with and may promote habitat protection and biodiversity, whether overtly or as a serendipitous by-product, depending on the CE parcels’ land use(s).

With the second-highest CE purpose for agriculture viability/livestock (over time, across the combined counties) and the dominant reason in six counties, our results affirm that LTs are employing CEs for farmland protection as a conservation priority (LTA, 2016). Using the full population of CEs (2823) held by numerous LTs over the decadal period in our chosen counties, our results expand Rissman and Merelender’s (2008) geographically localized finding and validate and generalize Rissman et al.’s (2007) finding that almost half of the 119 sample CEs were working landscape CEs. We qualify our results by the fact that use intensity ranges with use typology, and in some circumstances, working lands can co-exist with biological objectives from CE placement (Braza, 2017; Brunson & Huntsinger, 2008). Residential land use on a CE parcel can continue without defeating the CE purpose depending on parcel size and the landscape matrix (Farr et al., 2017; Rissman et al., 2007).

Our results may prompt the land conservation policy, conservation biology, and environmental planning communities to further examine the relationship between LUDs for tax appraisal on CE parcels and biodiversity protection objectives, especially since residential use could impact ecosystems (Hilty et al., 2006; Rissman et al., 2007) unless the parcels are sufficiently large or situated in a greater natural matrix (Farr et al., 2017). It is possible that those CEs that allow continued human land uses are being used to buffer rather than protect and restore tracts of pristine, core habitat areas or corridors. It is not clear whether the use(s) associated with the designated LUD will change over time on CE lands, warranting further examination given variability in levels and permissibility of activities on different CEs that may respond to or prevent them. If the CEs’ ultimate policy intent remains to conserve lands for ecosystem function (UCEA, 2007), our findings suggest that the intensity of LUDs for tax appraisal and the size of those parcels may impede this biodiversity objective (provided the deeds themselves do not limit allowed uses and also dependent on the landscape matrix composition (Lindenmayer, 2019)). The question becomes whether—regardless of explicit purpose—the CEs are promoting biodiversity protection objectives on these LUDs, and the extent to which the appraisals intensify and contribute to further conversion through property taxation that realize societal land use goals (Hilty et al., 2006; Rissman et al., 2007; Brandt, 2014; Owley & Rissman, 2016; IPCC, 2019). As a private land conservation policy mechanism seeking to balance competing social and ecological needs, the CE is nuanced, contextual, and layered, involving many assessed land uses, and we encourage further examination of its efficacy in changing land conversion, contributing to private land conservation categorization, and in realizing biodiversity protection.

## Supplementary Material

Appendix S1

Appendix S2

Appendix S3

Appendix S4

Appendix S5

Appendix S6

Appendix S7

Data S1

## Figures and Tables

**TABLE 1:** Poisson regression results for residential and agricultural CE counts versus total CEs

Assessment level over time		Residential CE counts		Agricultural CE counts	
	County	b_1s estimate	95% credible interval	b_1s estimate	95% credible interval
Within county	Albemarle, VA	0.02	(0.01, 0.031)^*^	0.077	(−0.051, 0.197)
Within county	Boulder, CO	0.035	(0.006, 0.066)^*^	−0.002	(−0.019, 0.014)
Within county	Charleston, SC	0.132	(0.121, 0.143)^*^	0.151	(0.128, 0.175)^*^
Within county	Douglas, MN	0.061	(−0.056, 0.185)	−0.016	(−0.057, 0.026)
Within county	Greenville, SC	0.195	(0.144, 0.244)^*^	−0.087	(−0.133, −0.039)^*^
Within county	Lebanon, PA	−0.147	(−0.283, −0.008)^*^	0.013	(−0.017, 0.045)
Within county	Loudoun, VA	0.081	(0.072, 0.089)^*^	−0.09	(−0.128, −0.052)^*^
Within county	Mesa, CO	0.038	(−0.036, 0.113)	0.053	(0.036, 0.07)^*^
Within county	Sacramento, CA	0.236	(0.137, 0.334)^*^	0.011	(−0.033, 0.011)
Within county	Sonoma, CA	0.046	(−0.014, 0.111)	0.035	(0.008, 0.063)^*^
Within county	Washington, MN	0.045	(0.035, 0.056)^*^	−0.23	(−0.258, −0.201)^*^
Within county	York, PA	0.062	(0.047, 0.077)^*^	−0.027	(−0.037, −0.017)^*^
*Across counties*	*All counties*	0.029	(−*0.009, 0.080*)	–*0.029*	(–*0.106, 0.045*)

* (denotes significance).

**TABLE 2:** Pre- and post-LUD statistics for the subset of CE parcels with *both values.*

County	Parcels where LUD changed post CE placement (%)	Fisher’s exact test statistic for relationship between pre and post-LUD	Monte carlo sig.	Pre-CE dominant LUD	Pre-CE top 3 LUDs	Pre-N	Post-CE dominant LUD	Post-CE top 3 LUDs	Post-N	Total *N*
Albemarle	6.47%	700.673	<.0001		Vacant residential^a^	378		Vacant residential^a^	366	665
				Res.	Residential/SF l–4^a^	281	Res.	Residential/SF 1–4^a^	287	
					Agriculture/Misc. Ag	5		Agriculture/Misc. Ag.	7	
Boulder	31.97%	1866.22	<.0001		Agriculture/Crops^a^	114		Public Govť ans Utilities/County	113	513
				Ag.	Miscellaneous	54	Pub. G	Agriculture/Crops^a^	93	
					Industrial/Mining	53		Residential/SF 1–4	53	
Charleston	23.02%	396.04	<.0001		Residential/SF l–4^a^	123		Agriculture^a^	113	265
				Res.	Agriculture^a^	99	Res.	Residential/SF 1–4^a^	111	
					Vacant residential	17		Vacant residential	11	
Douglas	25%	140.79	<.0001		Agriculture^a^	98		Agriculture+	79	112
				Ag.	Agriculture/Pasture	5	Ag.	Miscellaneous/Misc. Riparian	14	
					Residential/SF 1–4	4		Agriculture/Pasture and Residential/SF 1–4	5	
Greenville	29.59%	226.55	<.0001		Vacant residential^a^	44		Vacant residential^a^	45	98
				Res./Ag.	Vacant agriculture^a^	33	Res.	Agriculture/Industrial agriculture	19	
					Agriculture/Industrial Agriculture	11	Res.	Vacant agriculture^a^	16	
Lebanon	27.13%	215.162	<.0001		Agriculture/Misc. Ag^a^	91		Agriculture/Misc. Ag^a^	92	129
				Ag.	Miscellaneous	17	Ag.	Vacant/Misc. vacant	22	
					Residential	7		Agriculture/Crops	8	
Loudoun	67.66%	296.99	< .0001		Agriculture/Misc. Ag^a^	134		Vacant/Misc. Vacant^a^	140	269
				Ag.	Residential/SF l–4^a^	95	Vac.	Vacant residential^a^	72	
					Vacant/Misc. Vacant	25		Residential/SF 1–4^a^	39	
Mesa	3.18%	419.24	<.0001		Agriculture/Pasture^a^	139		Agriculture/Pasture^a^	133	227
				Ag.	Agriculture/Misc. Ag^a^	68	Ag.	Agriculture/Misc. Ag^a^	68	
					Agriculture/Orchard	12		Agriculture/Orchard	12	
Sacramento	3.51%	299.83	<.0001		Agriculture/Pasture^a^	99		Agriculture/Pasture^a^	99	114
				Ag.	Agriculture/Crops	6	Ag.	Agriculture/Crops	6	
					Public/Govť and Utilities/County	2		Public/Govť and Utilities/County	2	
Sonoma	6.12%	463.05	<.0001		Agriculture/Timber/Forest^a^	16		Agriculture/Vineyard^a^	18	98
				Ag.	Agriculture/Pasture^a^	15	Ag.	Agriculture/Timber/Forest^a^	15	
					Agriculture/Vineyard^a^	15	Ag.	Agriculture/Pasture and Agriculture/Preserve^a^	14	
Washington	64.95%	319.91	<.0001		Agriculture^a^	114		Residential^a^	134	194
				Ag.	Residential/SF 1–4	31	Res.	Residential/SF 1–4	22	
				Ag.	Residential^a^	30	Res.	Misc. Institutional	15	
York	6%	4551.3	<.0001	Ag.	Agriculture/Crops^a^	331	Ag.	Agriculture/Crops^a^ Residential/SF l–4^a^ Vacant agriculture^a^	298	883
					Residential/SF l–4^a^	213		Residential/SF l–4^a^	221	
					Vacant agriculture^a^	115		Vacant agriculture^a^	131	

a Indicates pre- and post-LUD pairs that comprise more than 10% of total CEs, regardless of whether the land uses match (pre- and post-CE placement).

**TABLE 3:** Comparison of LUD statistics for the two parcel subsets (pre- and post-CE, and only post-CE).

	For parcels with pre- and post-CE land use data				For parcels without pre CE-land use data, only post CE data			
		
County^a^	Post-CE dominant LUD	Post-CE top 3 LUDs	*N*	Total *N*	Mean parcel size ha. (SD)	Post-CE dominant LUD	Top 3 LUDs	*N*	Total *N*	Mean parcel size ha. (SD)
Albemarle	Res.	Vacant/Vacant residential	366	665	34.10(122.13)		Vacant/Vacant residential	119	183	32.65 (129.37)
		Residential/SF 1–4	287			Res.	Residential/SF 1–4	60		
		Agriculture/Misc. Ag.	7				Agriculture/Misc. Ag	3		
Boulder	Pub. G.	Public Govť and Utilities/County	113	513	23.33 (99.74)	Ag.	Agriculture/Crops	79	289	17.94 (88.45)
		Agriculture/Crops	93				Agriculture/Pasture	61		
		Residential/SF 1–4	53				Residential/SF 1–4	38		
Charleston	Res.	Agriculture/Agriculture	113	265	54.94 (318.62)	Res.	Residential/SF 1–4	499	620	3.35 (50.94)
		Residential/SF 1–4	111				Vacant/Misc. Vacant	75		
		Vacant/Vacant Residential	11				Agriculture/Agriculture	20		
Greenville	Res.	Vacant/Vacant residential	45	98	138.85 (2035.53)	Res.	Vacant/Vacant residential	143	172	5.87 (64.44)
		Agriculture/Industrial agriculture	19				Residential/SF 1–4	10		
		Vacant/Vacant agriculture	16				Residential/Misc. residential	8		
Lebanon	Ag.	Agriculture/Misc. Ag	92	129	34.07 (50.87)	Ag.	Agriculture/Misc. Ag	7	8	39.59 (21.38)
		Vacant/Misc. Vacant	22				Agriculture/Crops	1		
		Agriculture/Crops	8							
Loudoun	Vac.	Vacant/Misc. Vacant	140	269	24.68 (84.28)	Res.	Vacant/Vacant residential	1365	2126	4.29 (29.57)
		Vacant/Vacant residential	72				Residential/Misc. Res	355		
		Residential/SF 1–4	39				Residential/Misc. Res	285		
Mesa	Ag.	Agriculture/Pasture	133	227	92.85 (919.26)	Ag.	Agriculture/Pasture	28	63	96.51 (672.96)
		Agriculture/Misc. Ag	68				Agriculture/Misc. Ag	23		
		Agriculture/Orchard	12				Agriculture/Orchard	5		
Sacramento	Ag.	Agriculture/Pasture	99	114	142.30 (270.58)	Ag.	Agriculture/Pasture	33	63	47.85 (172.46)
		Agriculture/Crops	6				Miscellaneous/Misc. Riparian	7		
		Public/Gov’t and Utilities/County	2				Residential/Misc. residential	6		
Sonoma	Ag.	Agriculture/Vineyard	18	98	51.44 (168.34)	Ag.	Agriculture/Pasture	83	199	64.43 (230.66)
		Agriculture/Timber/Forest	15			Ag.	Agriculture/Timber/Forest	31		
		Agriculture/Pasture and Agriculture/Preserve	14				Agriculture/Vineyard	26		
Washington	Res.	Residential/Residential	134	194	2.38 (13.17)	Res.	Residential/Residential	183	422	2.70 (12.39)
		Residential/SF 1–4	22				Residential/SF 1–4	133		
		Misc. Institutional	15				Miscellaneous/Exempt	70		
York	Ag.	Agriculture/Crops	298	883	21.63 (72.01)	Ag.	Residential/SF 1–4	33	110	22.96 (96.43)
		Residential/SF 1–4	221				Agriculture/Crops	24		
		Vacant/Vacant agriculture	131				Vacant/Vacant agriculture	17		

a Douglas does not have any post-only data.

**TABLE 4:** Dominant land use designations (LUDs) and chi-square results for the correspondence of primary LUD and first CE reason for parcels with a CE in a given year (bold county name denotes significant association).

US census region	State	County	Residential or agriculturally-dominant LUD	First listed CE reason by highest percentage	Chi-square *p*-value for LUD by CE reason	Significant association (or Independence) between LUD and CE reason (*p* <.05)	Largest mean CE parcel size in any given year (ha)	CE parcel median range over time (ha)
South	VA	Albemarle	Residential	IRS code 26 U.S.C. 170 (h) (4): pursuant to a clearly delineated governmental conservation policy^a^|42.26% (167 out of 361 CEs)	0.9804	Not significant |independent	56.66	12.11–28.82
	VA	**Loudon**	Residential	Open space preservation 159.82% (271 out of 453 CEs)	4.25E-15	**Significant** |dependent	12.29	0.54–7.78
South	SC	Charleston	Residential	IRS code 26 U.S.C. 170 (h) (4): the protection of a relatively natural habitat of fish, wildlife, or plants^a^|64.19% (138 out of 215 CEs)	0.0862	Not significant |independent	16.24	0.14–0.29
	sc	**Greenville**	Residential	Natural condition/natural environment/natural resource values|22.41% (26 out of 116 CEs)	3.90E-05	**Significant** |dependent	196.38	0.07–6.57
Midwest	MN	Douglas	Agricultural	Soil|65.62% (42 out of 64 CEs)	0.2886	Not significant |independent	30.14	16.88–29.95
	MN	**Washington**	Residential	Scenic value/beauty|49.41% (126 out of 255 CEs)	2.29E-35	**Significant** |dependent	4.79	0.15–1.30
Northeast	PA	Lebanon	Agricultural	Agricultural viability/livestock|84.68% (94 out of 111 CEs)	0.7261	Not significant |independent	47.46	24.09–44.31
	PA	**York**	Agricultural	Agricultural viability/livestock|61.69% (190 out of 308 CEs)	4.33E-23	**Significant** |dependent	44.50	10.12–36.84
West	CA	Sacramento	Agricultural	Agricultural viability/livestock |21.43% (15 out of 70 CEs)	0.7829	Not significant |independent	124.89	37.26–81.61
	CA	Sonoma	Agricultural	Open space preservation|48% (60 out of 125 CEs)	0.0551	Not significant |independent	69.58	14.30–29.06
West	CO	**Boulder**	Agricultural	Agricultural viability/livestock|19.79% (111 out of 561 CEs)	1.61E-13	**Significant** |dependent	37.08	7.64–14.10
	CO	**Mesa**	Agricultural	Agricultural viability/livestock|41.85% (77 out of 184 CEs)	0.0007	**Significant** |dependent	142.62	20.80–65.33
**All counties**			Agricultural (1997–1998)|Residential (1999–2009)	Open space preservation|20.30% (573 out of 2823 CEs) [note: Agricultural viability/livestock|18.53% (523 out of 2823 CEs)]	5.09E-92	**Significant** |dependent	25.24	0.26–3.77

a Indicates a biological reason.

## Data Availability

These data will initially be made accessible, where county agreements do not preclude sharing, via a request to the authors. Within a year from publication, the datasets will be made available through a portal at Clemson University.
