# Supplementary material for: Unpacking conservation easements’ assessed land use designations and their implications for realizing biodiversity protection
Source: Conserv Sci Pract. Author manuscript; Available in PMC 2025 Jun 1. (PMC11675600; doi:10.1111/csp2.13130)
Supplement: Appendix S1 [file NIHMS1988779-supplement-Appendix_S1.docx]

**Appendix S1: Mapping Table with Master Land Use Designations**

| **General Land Use** | **Intermediate** | **Specific Land Use** | **Master Code** | **MLUD_Classification** |
| --- | --- | --- | --- | --- |
| Residential |  |  | 1000 | Residential |
|  | SF 1-4 |  | 1100 | Residential; SF 1-4 |
|  |  | single family dwelling | 1101 | Residential; SF 1-4; single family dwelling |
|  |  | condominium | 1102 | Residential; SF 1-4; condominium |
|  |  | [ADDITIONAL CATEGORIES NOT DISPLAYED] | | |
|  | MF - 5 or more |  | 1200 | MF - 5 or more |
|  |  | 5-10 units/apartment structure | 1201 | MF - 5 or more; 5-10 units/apartment structure |
|  |  | 5-10 units/2 or more structures | 1202 | MF - 5 or more; 5-10 units/2 or more structures |
|  |  | [ADDITIONAL CATEGORIES NOT DISPLAYED] | | |
|  | PUD |  | 1300 | Residential; PUD |
|  | Rural Res |  | 1400 | Residential; Rural Res |
|  |  | rural residential home site | 1401 | Residential; Rural Res; rural residential home site |
|  |  | [ADDITIONAL CATEGORIES NOT DISPLAYED] | | |
|  | Lodging |  | 1500 | Residential; Lodging |
|  |  | motel/less than 50 units | 1501 | Residential; Lodging; motel/less than 50 units |
|  |  | [ADDITIONAL CATEGORIES NOT DISPLAYED] | | |
|  | Group Living |  | 1600 | Residential; Group Living |
|  |  | sorority or fraternity house | 1601 | Residential; Group Living ; sorority or fraternity house |
|  |  | [ADDITIONAL CATEGORIES NOT DISPLAYED] | | |
|  | Mobile home/Park |  | 1700 | Residential; Mobile home/Park |
|  |  | mobile home park | 1701 | Residential; Mobile home/Park; mobile home park |
|  |  | [ADDITIONAL CATEGORIES NOT DISPLAYED] | | |
|  | Misc Res |  | 1800 | Residential; Misc Res |
|  |  | common area with structures | 1801 | Residential; Misc Res; common area with structures |
|  |  | [ADDITIONAL CATEGORIES NOT DISPLAYED] | | |
| Retail & Commercial |  |  | 2000 | Retail & Commercial |
|  | Retail |  | 2100 | Retail & Commercial; Retail |
|  |  | single story store | 2101 | Retail & Commercial; Retail; single story store |
|  |  | [ADDITIONAL CATEGORIES NOT DISPLAYED] | | |
|  | Mixed Use |  | 2200 | Retail & Commercial; Mixed Use |
|  |  | mixed use (store with residential units) | 2201 | Retail & Commercial; Mixed Use; mixed use (store with residential units) |
|  |  | [ADDITIONAL CATEGORIES NOT DISPLAYED] | | |
|  | Shopping Center |  | 2300 | Retail & Commercial; Shopping Center |
|  |  | regional shopping center | 2301 | Retail & Commercial; Shopping Center; regional shopping center |
|  |  | [ADDITIONAL CATEGORIES NOT DISPLAYED] | | |
|  | Commercial |  | 2400 | Retail & Commercial; Commercial |
|  |  | service station | 2401 | Retail & Commercial; Commercial; service station |
|  |  | [ADDITIONAL CATEGORIES NOT DISPLAYED] | | |
|  | Food Service |  | 2500 | Retail & Commercial; Food Service |
|  |  | restaurant (dining) | 2501 | Retail & Commercial; Food Service; restaurant (dining) |
|  |  | [ADDITIONAL CATEGORIES NOT DISPLAYED] | | |
|  | Misc Commerical |  | 2600 | Retail & Commercial; Misc Commerical |
|  |  | commercial common area | 2601 | Retail & Commercial; Misc Commerical; commercial common area |
|  |  | [ADDITIONAL CATEGORIES NOT DISPLAYED] | | |
| Office |  |  | 3000 | Office |
|  | Office |  | 3100 | Office; Office |
|  |  | one story office building | 3101 | Office; Office ; one story office building |
|  |  | [ADDITIONAL CATEGORIES NOT DISPLAYED] | | |
|  | Misc Office |  | 3200 | Office; Misc Office |
|  |  | office common area | 3201 | Office; Misc Office; office common area |
|  |  | [ADDITIONAL CATEGORIES NOT DISPLAYED] | | |
| Recreational |  |  | 4000 | Recreational |
|  | Public Rec |  | 4100 | Recreational; Public Rec |
|  |  | skating rink (roller or ice) | 4101 | Recreational; Public Rec; skating rink (roller or ice) |
|  |  | [ADDITIONAL CATEGORIES NOT DISPLAYED] | | |
|  | Private Rec |  | 4200 | Recreational; Private Rec |
|  |  | private golf course | 4201 | Recreational; Private Rec; private golf course |
|  |  | [ADDITIONAL CATEGORIES NOT DISPLAYED] | | |
|  | Misc Recreational |  | 4300 | Recreational; Misc Recreational |
|  |  | Swimming Pool | 4301 | Recreational; Misc Recreational;Swimming Pool |
|  |  | [ADDITIONAL CATEGORIES NOT DISPLAYED] | | |
| Industrial |  |  | 5000 | Industrial |
|  | Industrial |  | 5100 | Industrial; Industrial |
|  |  | heavy industry | 5101 | Industrial; Industrial; heavy industry |
|  |  | [ADDITIONAL CATEGORIES NOT DISPLAYED] | | |
|  | Manufacturing |  | 5200 | Industrial; Manufacturing |
|  |  | packing plant | 5201 | Industrial; Manufacturing ; packing plant |
|  |  | [ADDITIONAL CATEGORIES NOT DISPLAYED] | | |
|  | Mixed Industrial |  | 5300 | Industrial; Mixed Industrial |
|  |  | light manufacturing and light industrial | 5301 | Industrial; Mixed Industrial ; light manufacturing and light industrial |
|  |  | [ADDITIONAL CATEGORIES NOT DISPLAYED] | | |
|  | Warehousing/Storage |  | 5400 | Industrial; Warehousing/Storage |
|  |  | warehousing/active | 5401 | Industrial; Warehousing/Storage; warehousing/active |
|  |  | [ADDITIONAL CATEGORIES NOT DISPLAYED] | | |
|  | Processing |  | 5500 | Industrial; Processing |
|  |  | building materials processing (e.g. lumber mills and specialty products) | 5501 | Industrial; Processing; building materials processing (e.g. lumber mills and specialty products) |
|  |  | [ADDITIONAL CATEGORIES NOT DISPLAYED] | | |
|  | Distribution |  | 5600 | Industrial; Distribution |
|  |  | building materials distribution (e.g. lumber yards) | 5601 | Industrial; Distribution; building materials distribution (e.g. lumber yards) |
|  |  | [ADDITIONAL CATEGORIES NOT DISPLAYED] | | |
|  | Mining |  | 5700 | Industrial; Mining |
|  |  | mining (sand, gravel, clay, gas well, shale, etc.) | 5701 | Industrial; Mining; mining (sand, gravel, clay, gas well, shale, etc.) |
|  |  | [ADDITIONAL CATEGORIES NOT DISPLAYED] | | |
|  | Misc Industrial |  | 5800 | Industrial; Misc Industrial |
|  |  | airport (private) | 5801 | Industrial; Misc Industrial; airport (private) |
|  |  | [ADDITIONAL CATEGORIES NOT DISPLAYED] | | |
|  | Oil and Gas |  | 5900 | Industrial; Oil and Gas |
|  |  | Producing Oil (Primary or Secondary) | 5901 | Industrial; Oil and Gas; Producing Oil (Primary or Secondary) |
|  |  | [ADDITIONAL CATEGORIES NOT DISPLAYED] | | |
| Agriculture |  |  | 6000 | Agriculture |
|  | Industrial Ag |  | 6100 | Agriculture; Industrial Ag |
|  |  | feed lot | 6101 | Agriculture; Industrial Ag; feed lot |
|  |  | [ADDITIONAL CATEGORIES NOT DISPLAYED] | | |
|  | Orchard |  | 6200 | Agriculture; Orchard |
|  |  | irrigated or dry orchard (pear, walnut, olive, peach, cherry, orange, almond, plum, prune, kiwi, apple, etc.) | 6201 | Agriculture; Orchard; irrigated or dry orchard (pear, walnut, olive, peach, cherry, orange, almond, plum, prune, kiwi, apple, etc.) |
|  |  | [ADDITIONAL CATEGORIES NOT DISPLAYED] | | |
|  | Vineyard |  | 6300 | Agriculture; Vineyard |
|  |  | irrigated or dry vineyard (whether premium varietals or not, and includes vines and bush fruit) | 6301 | Agriculture; Vineyard; irrigated or dry vineyard (whether premium varietals or not, and includes vines and bush fruit) |
|  |  | [ADDITIONAL CATEGORIES NOT DISPLAYED] | | |
|  | Crops |  | 6400 | Agriculture; Crops |
|  |  | irrigated truck (row) crops | 6401 | Agriculture; Crops; irrigated truck (row) crops |
|  |  | [ADDITIONAL CATEGORIES NOT DISPLAYED] | | |
|  | Pasture |  | 6500 | Agriculture; Pasture |
|  |  | irrigated or dry pasture | 6501 | Agriculture; Pasture; irrigated or dry pasture |
|  |  | [ADDITIONAL CATEGORIES NOT DISPLAYED] | | |
|  | Equestrian |  | 6600 | Agriculture; Equestrian |
|  |  | horse ranch (with or without residence or manufactured home) | 6601 | Agriculture; Equestrian; horse ranch (with or without residence or manufactured home) |
|  |  | [ADDITIONAL CATEGORIES NOT DISPLAYED] | | |
|  | Timber/Forest |  | 6700 | Agriculture; Timber/Forest |
|  |  | timber land (redwood, fir, mixture, etc.) with or without residence or manufactured home | 6701 | Agriculture; Timber/Forest ; timber land (redwood, fir, mixture, etc.) with or without residence or manufactured home |
|  |  | [ADDITIONAL CATEGORIES NOT DISPLAYED] | | |
|  | Preserve |  | 6800 | Agriculture; Preserve |
|  |  | ag preserve/timber preserve | 6801 | Agriculture; Preserve; ag preserve/timber preserve |
|  | Misc Ag |  | 6900 | Agriculture; Misc Ag |
|  |  | specialty farm | 6901 | Agriculture; Misc Ag; specialty farm |
|  |  | [ADDITIONAL CATEGORIES NOT DISPLAYED] | | |
| Institutional Properties |  |  | 7000 | Institutional Properties |
|  | Religious Buildings |  | 7100 | Institutional Properties; Religious Buildings |
|  |  | Church (exempt) | 7101 | Institutional Properties; Religious Buildings; Church (exempt) |
|  |  | [ADDITIONAL CATEGORIES NOT DISPLAYED] | | |
|  | Schools |  | 7200 | Institutional Properties; Schools |
|  |  | private schools | 7201 | Institutional Properties; Schools; private schools |
|  |  | [ADDITIONAL CATEGORIES NOT DISPLAYED] | | |
|  | Medical |  | 7300 | Institutional Properties; Medical |
|  |  | private social service agency (exempt, partially exempt, non-exempt) | 7301 | Institutional Properties; Medical ; private social service agency (exempt, partially exempt, non-exempt) |
|  |  | [ADDITIONAL CATEGORIES NOT DISPLAYED] | | |
|  | Institutional Residential |  | 7400 | Institutional Properties; Institutional Residential |
|  |  | retirement home | 7401 | Institutional Properties; Institutional Residential ; retirement home |
|  |  | [ADDITIONAL CATEGORIES NOT DISPLAYED] | | |
|  | Cemeteries/Mortuaries |  | 7500 | Institutional Properties; Cemeteries/Mortuaries |
|  |  | cemetery (and zero-valued property) | 7501 | Institutional Properties; Cemeteries/Mortuaries; cemetery (and zero-valued property) |
|  |  | [ADDITIONAL CATEGORIES NOT DISPLAYED] | | |
|  | Misc Institutional |  | 7600 | Institutional Properties; Misc Institutional |
|  |  | volunteer fire department | 7601 | Institutional Properties; Misc Institutional ; volunteer fire department |
|  |  | [ADDITIONAL CATEGORIES NOT DISPLAYED] | | |
| Public/Gov't & Utilities |  |  | 8000 | Public/Gov't & Utilities |
|  | Utility |  | 8100 | Public/Gov't & Utilities; Utility |
|  |  | SBE property/utility (exempt, partially or non-exempt) | 8101 | Public/Gov't & Utilities; Utility; SBE property/utility (exempt, partially or non-exempt) |
|  |  | [ADDITIONAL CATEGORIES NOT DISPLAYED] | | |
|  | Federal |  | 8200 | Public/Gov't & Utilities; Federal |
|  |  | federal property (exempt, partially or non-exempt), including buildings, military installations, etc. | 8201 | Public/Gov't & Utilities; Federal; federal property (exempt, partially or non-exempt), including buildings, military installations, etc. |
|  |  | [ADDITIONAL CATEGORIES NOT DISPLAYED] | | |
|  | State |  | 8300 | Public/Gov't & Utilities; State |
|  |  | state property (exempt, partially or non-exempt), including buildings, lands, parks, colleges, schools, hospitals, etc. | 8301 | Public/Gov't & Utilities; State; state property (exempt, partially or non-exempt), including buildings, lands, parks, colleges, schools, hospitals, etc. |
|  |  | [ADDITIONAL CATEGORIES NOT DISPLAYED] | | |
|  | County |  | 8400 | Public/Gov't & Utilities; County |
|  |  | county property (exempt, partially or non-exempt), including land, buildings, parks, airport, hospitals, water agencies, etc. | 8401 | Public/Gov't & Utilities; County; county property (exempt, partially or non-exempt), including land, buildings, parks, airport, hospitals, water agencies, etc. |
|  |  | [ADDITIONAL CATEGORIES NOT DISPLAYED] | | |
|  | City |  | 8500 | Public/Gov't & Utilities; City |
|  |  | city property (exempt, partially or non-exempt), including land, buildings, parks, garages, parking lots, airports, etc. | 8501 | Public/Gov't & Utilities; City; city property (exempt, partially or non-exempt), including land, buildings, parks, garages, parking lots, airports, etc. |
|  |  | [ADDITIONAL CATEGORIES NOT DISPLAYED] | | |
|  | Special District |  | 8600 | Public/Gov't & Utilities; Special District |
|  |  | public school district property or possessory interest | 8601 | Public/Gov't & Utilities; Special District; public school district property or possessory interest |
|  |  | [ADDITIONAL CATEGORIES NOT DISPLAYED] | | |
|  | Misc Gov't Property |  | 8700 | Public/Gov't & Utilities; Misc Gov't Property |
|  |  | Public Housing (exempt, partially exempt) | 8701 | Public/Gov't & Utilities; Misc Gov't Property; Public Housing (exempt, partially exempt) |
|  |  | [ADDITIONAL CATEGORIES NOT DISPLAYED] | | |
|  | Misc Partially Exempt |  | 8800 | Public/Gov't & Utilities; Misc Partially Exempt |
|  |  | Commercial - partially exempt | 8801 | Public/Gov't & Utilities; Misc Partially Exempt; Commercial - partially exempt |
|  |  | [ADDITIONAL CATEGORIES NOT DISPLAYED] | | |
| Vacant |  |  | 9000 | Vacant |
|  | Vacant Residential |  | 9100 | Vacant; Vacant Residential |
|  |  | vacant residential land undeveloped (whether problematic or not) | 9101 | Vacant; Vacant Residential ; vacant residential land undeveloped (whether problematic or not) |
|  |  | [ADDITIONAL CATEGORIES NOT DISPLAYED] | | |
|  | Vacant Retail/Comm |  | 9200 | Vacant; Vacant Retail/Comm |
|  |  | vacant retail/commercial land undeveloped | 9201 | Vacant; Vacant Retail/Comm; vacant retail/commercial land undeveloped |
|  |  | [ADDITIONAL CATEGORIES NOT DISPLAYED] | | |
|  | Vacant Indust |  | 9300 | Vacant; Vacant Indust |
|  |  | undeveloped industrial land | 9301 | Vacant; Vacant Indust; undeveloped industrial land |
|  |  | [ADDITIONAL CATEGORIES NOT DISPLAYED] | | |
|  | Vacant Office |  | 9400 | Vacant; Vacant Office |
|  |  | vacant office land undeveloped (whether problematic or not) | 9401 | Vacant; Vacant Office; vacant office land undeveloped (whether problematic or not) |
|  |  | [ADDITIONAL CATEGORIES NOT DISPLAYED] | | |
|  | Vacant Gov't |  | 9500 | Vacant; Vacant Gov't |
|  |  | vacant federal land | 9501 | Vacant; Vacant Gov't; vacant federal land |
|  |  | [ADDITIONAL CATEGORIES NOT DISPLAYED] | | |
|  | Vacant Ag |  | 9600 | Vacant; Vacant Ag |
|  | Vacant Rec |  | 9700 | Vacant; Vacant Rec |
|  |  | vacant recreational land undeveloped | 9701 | Vacant; Vacant Rec; vacant recreational land undeveloped |
|  |  | vacant recreational land with utilities | 9702 | Vacant; Vacant Rec; vacant recreational land with utilities |
|  | Vacant Institutional |  | 9800 | Vacant; Vacant Institutional |
|  |  | health care | 9801 | Vacant; Vacant Institutional; health care |
|  | Misc Vacant |  | 9900 | Vacant; Misc Vacant |
|  |  | < 1.0 Acre | 9901 | Vacant; Misc Vacant; < 1.0 Acre |
|  |  | [ADDITIONAL CATEGORIES NOT DISPLAYED] | | |
| Miscellaneous |  |  | 10000 | Miscellaneous |
|  | Misc Non-Possessory Rights |  | 10100 | Miscellaneous; Misc Non-Possessory Rights |
|  |  | other extractable commodity rights (stream rights, power plant, etc.) | 10101 | Miscellaneous; Misc Non-Possessory Rights; other extractable commodity rights (stream rights, power plant, etc.) |
|  |  | [ADDITIONAL CATEGORIES NOT DISPLAYED] | | |
|  | Misc Riparian |  | 10200 | Miscellaneous; Misc Riparian |
|  |  | wells or tank sites, spring/other water sources | 10201 | Miscellaneous; Misc Riparian; wells or tank sites, spring/other water sources |
|  |  | [ADDITIONAL CATEGORIES NOT DISPLAYED] | | |
|  | Misc Land |  | 10300 | Miscellaneous; Misc Land |
|  |  | park, greenbelt, etc. | 10301 | Miscellaneous; Misc Land; park, greenbelt, etc. |
|  |  | [ADDITIONAL CATEGORIES NOT DISPLAYED] | | |
|  | Misc General |  | 10400 | Miscellaneous; Misc General |
|  |  | unsecured uses (possessory interests, unsecured buildings, etc.) | 10401 | Miscellaneous; Misc General; unsecured uses (possessory interests, unsecured buildings, etc.) |
|  |  | [ADDITIONAL CATEGORIES NOT DISPLAYED] | | |
|  | Exempt |  | 10500 | Miscellaneous; Exempt |
|  |  | Other | 10501 | Miscellaneous; Exempt; Other |
|  | Personal Property |  | 10600 | Miscellaneous; Personal Property |
