# Supplementary material for: Unpacking conservation easements’ assessed land use designations and their implications for realizing biodiversity protection
Source: Conserv Sci Pract. Author manuscript; Available in PMC 2025 Jun 1. (PMC11675600; doi:10.1111/csp2.13130)
Supplement: Appendix S2 [file NIHMS1988779-supplement-Appendix_S2.docx]

**Appendix S2: One-Way ANOVA Results**

**One-Way ANOVA All Counties Parc Hectares**

Df Sum Sq Mean Sq F value Pr(*>*F)

| county ID | 11 | 3165726 | 287793 | 23.17 | 0.0000 |
| --- | --- | --- | --- | --- | --- |
| Residuals | 8596 | 106447749 | 12422 |  |  |

**Tukey Multiple Comparisons of Means, 95% CI, All Counties Parc Hectares**

*Fit* : *aov*(*formula* = *Parc Hectares∼* County, data = group cnty yr size)

| **Counties** | **diff** | **lwr** | **upr** | **p adj** |
| --- | --- | --- | --- | --- |
| Boulder-Albemarle | -12.8 | -32.0 | 6.5 | 0.57857 |
| Charleston-Albemarle | -11.3 | -29.7 | 7.1 | 0.68928 |
| Douglas-Albemarle | -5.3 | -42.3 | 31.7 | 1.00000 |
| Greenville-Albemarle | 66.8 | 32.6 | 101.1 | 0.00000 |
| Lebanon-Albemarle | 2.1 | -32.0 | 36.1 | 1.00000 |
| Loudoun-Albemarle | -24.9 | -39.7 | -10.1 | 0.00000 |
| Mesa-Albemarle | 41.7 | 17.8 | 65.5 | 0.00000 |
| Sacramento-Albemarle | 49.5 | 18.7 | 80.3 | 0.00001 |
| Sonoma-Albemarle | 22.4 | 0.4 | 44.3 | 0.04085 |
| Washington-Albemarle | -29.3 | -48.8 | -9.9 | 0.00005 |
| York-Albemarle | -13.6 | -30.9 | 3.6 | 0.29048 |
| Charleston-Boulder | 1.5 | -18.2 | 21.2 | 1.00000 |
| Douglas-Boulder | 7.5 | -30.2 | 45.2 | 0.99997 |
| Greenville-Boulder | 79.6 | 44.6 | 114.6 | 0.00000 |
| Lebanon-Boulder | 14.8 | -19.9 | 49.6 | 0.96512 |
| Loudoun-Boulder | -12.2 | -28.6 | 4.2 | 0.38494 |
| Mesa-Boulder | 54.4 | 29.5 | 79.3 | 0.00000 |
| Sacramento-Boulder | 62.3 | 30.6 | 93.9 | 0.00000 |
| Sonoma-Boulder | 35.1 | 12.1 | 58.2 | 0.00004 |
| Washington-Boulder | -16.6 | -37.3 | 4.1 | 0.26850 |
| York-Boulder | -0.9 | -19.5 | 17.8 | 1.00000 |
| Douglas-Charleston | 6.0 | -31.2 | 43.2 | 1.00000 |
| Greenville-Charleston | 78.1 | 43.7 | 112.6 | 0.00000 |
| Lebanon-Charleston | 13.4 | -20.9 | 47.6 | 0.98238 |
| Loudoun-Charleston | -13.6 | -29.0 | 1.7 | 0.13702 |
| Mesa-Charleston | 53.0 | 28.8 | 77.2 | 0.00000 |
| Sacramento-Charleston | 60.8 | 29.7 | 91.9 | 0.00000 |
| Sonoma-Charleston | 33.7 | 11.4 | 56.0 | 0.00005 |
| Washington-Charleston | -18.0 | -37.9 | 1.8 | 0.11762 |
| York-Charleston | -2.3 | -20.0 | 15.4 | 1.00000 |
| Greenville-Douglas | 72.1 | 25.0 | 119.2 | 0.00004 |
| Lebanon-Douglas | 7.3 | -39.6 | 54.3 | 1.00000 |
| Loudoun-Douglas | -19.7 | -55.3 | 15.9 | 0.81526 |
| Mesa-Douglas | 46.9 | 6.7 | 87.2 | 0.00760 |
| Sacramento-Douglas | 54.8 | 10.1 | 99.5 | 0.00359 |
| Sonoma-Douglas | 27.6 | -11.5 | 66.8 | 0.46822 |
| Washington-Douglas | -24.0 | -61.8 | 13.7 | 0.63601 |
| York-Douglas | -8.3 | -45.0 | 28.3 | 0.99986 |
| Lebanon-Greenville | -64.8 | -109.6 | -20.0 | 0.00015 |
| Loudoun-Greenville | -91.8 | -124.5 | -59.1 | 0.00000 |
| Mesa-Greenville | -25.2 | -62.9 | 12.5 | 0.56199 |
| Sacramento-Greenville | -17.3 | -59.8 | 25.1 | 0.97452 |
| Sonoma-Greenville | -44.5 | -81.0 | -8.0 | 0.00394 |
| Washington-Greenville | -96.2 | -131.2 | -61.1 | 0.00000 |
| York-Greenville | -80.5 | -114.3 | -46.6 | 0.00000 |
| Loudoun-Lebanon | -27.0 | -59.5 | 5.5 | 0.21792 |
| Mesa-Lebanon | 39.6 | 2.1 | 77.1 | 0.02778 |
| Sacramento-Lebanon | 47.4 | 5.2 | 89.7 | 0.01304 |
| Sonoma-Lebanon | 20.3 | -16.0 | 56.6 | 0.80214 |
| Washington-Lebanon | -31.4 | -66.2 | 3.5 | 0.12586 |
| York-Lebanon | -15.7 | -49.3 | 18.0 | 0.93476 |
| Mesa-Loudoun | 66.6 | 45.0 | 88.2 | 0.00000 |
| Sacramento-Loudoun | 74.4 | 45.4 | 103.5 | 0.00000 |
| Sonoma-Loudoun | 47.3 | 27.9 | 66.7 | 0.00000 |
| Washington-Loudoun | -4.4 | -21.0 | 12.2 | 0.99941 |
| York-Loudoun | 11.3 | -2.6 | 25.2 | 0.24782 |
| Sacramento-Mesa | 7.8 | -26.8 | 42.4 | 0.99987 |
| Sonoma-Mesa | -19.3 | -46.3 | 7.7 | 0.45017 |
| Washington-Mesa | -71.0 | -96.0 | -46.0 | 0.00000 |
| York-Mesa | -55.3 | -78.6 | -31.9 | 0.00000 |
| Sonoma-Sacramento | -27.1 | -60.4 | 6.2 | 0.24427 |
| Washington-Sacramento | -78.8 | -110.5 | -47.1 | 0.00000 |
| York-Sacramento | -63.1 | -93.5 | -32.7 | 0.00000 |
| Washington-Sonoma | -51.7 | -74.9 | -28.5 | 0.00000 |
| York-Sonoma | -36.0 | -57.3 | -14.6 | 0.00000 |
| York-Washington | 15.7 | -3.1 | 34.5 | 0.21016 |
