# Supplementary figures and images for: Unpacking conservation easements’ assessed land use designations and their implications for realizing biodiversity protection
Source: Conserv Sci Pract. Author manuscript; Available in PMC 2025 Jun 1. (PMC11675600; doi:10.1111/csp2.13130)

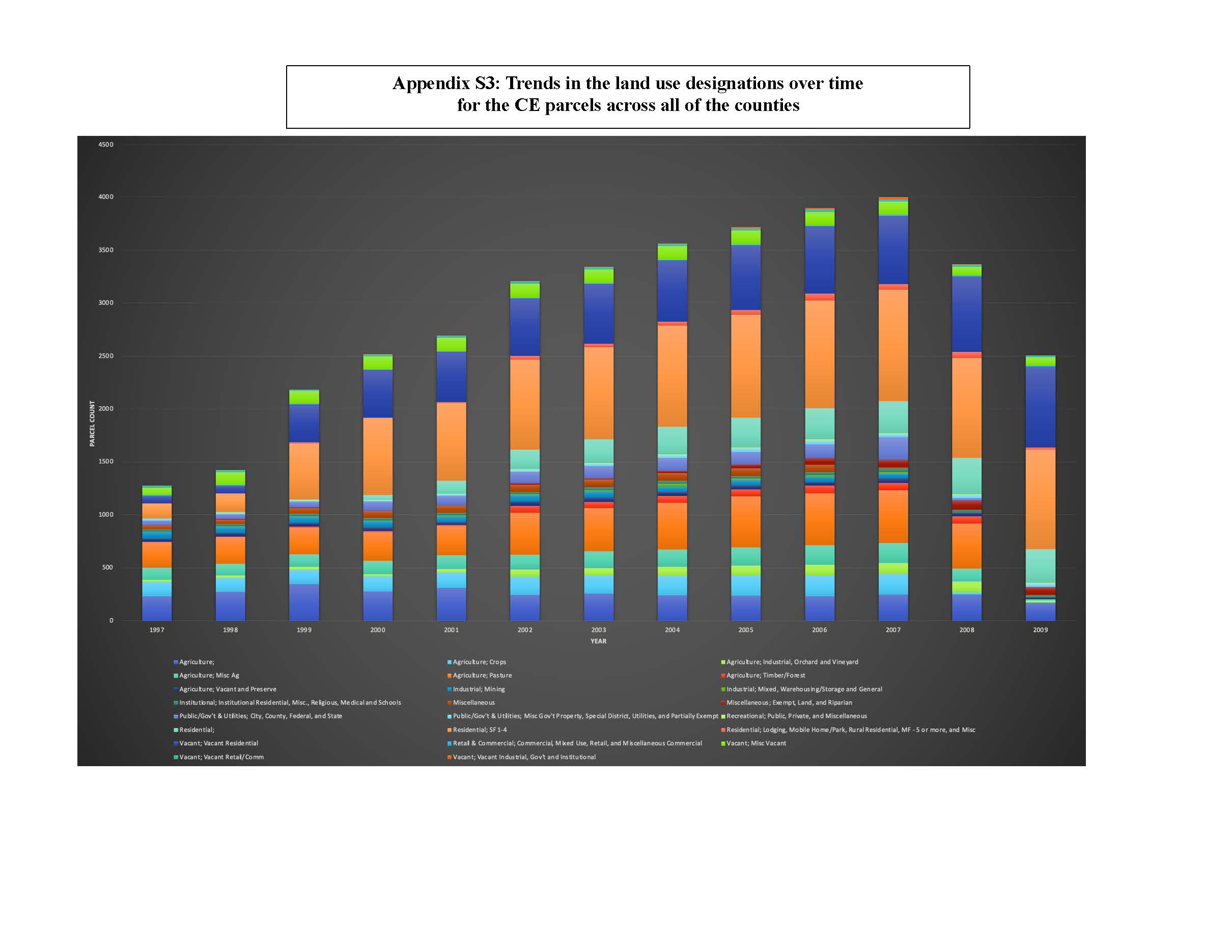

Supplement: Appendix S3 [file NIHMS1988779-supplement-Appendix_S3.jpg]

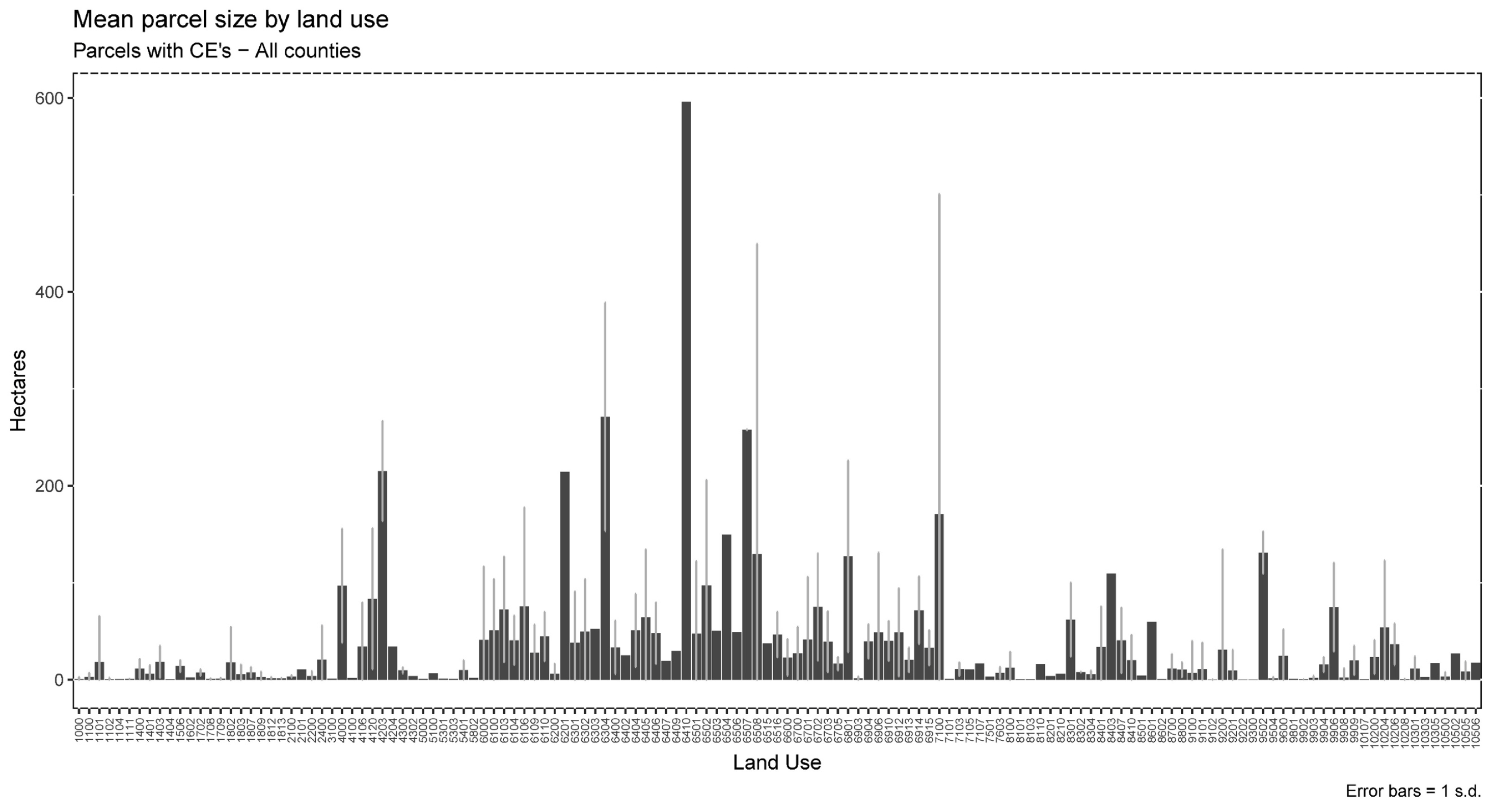

Supplement: Appendix S5 [file NIHMS1988779-supplement-Appendix_S5.tiff]

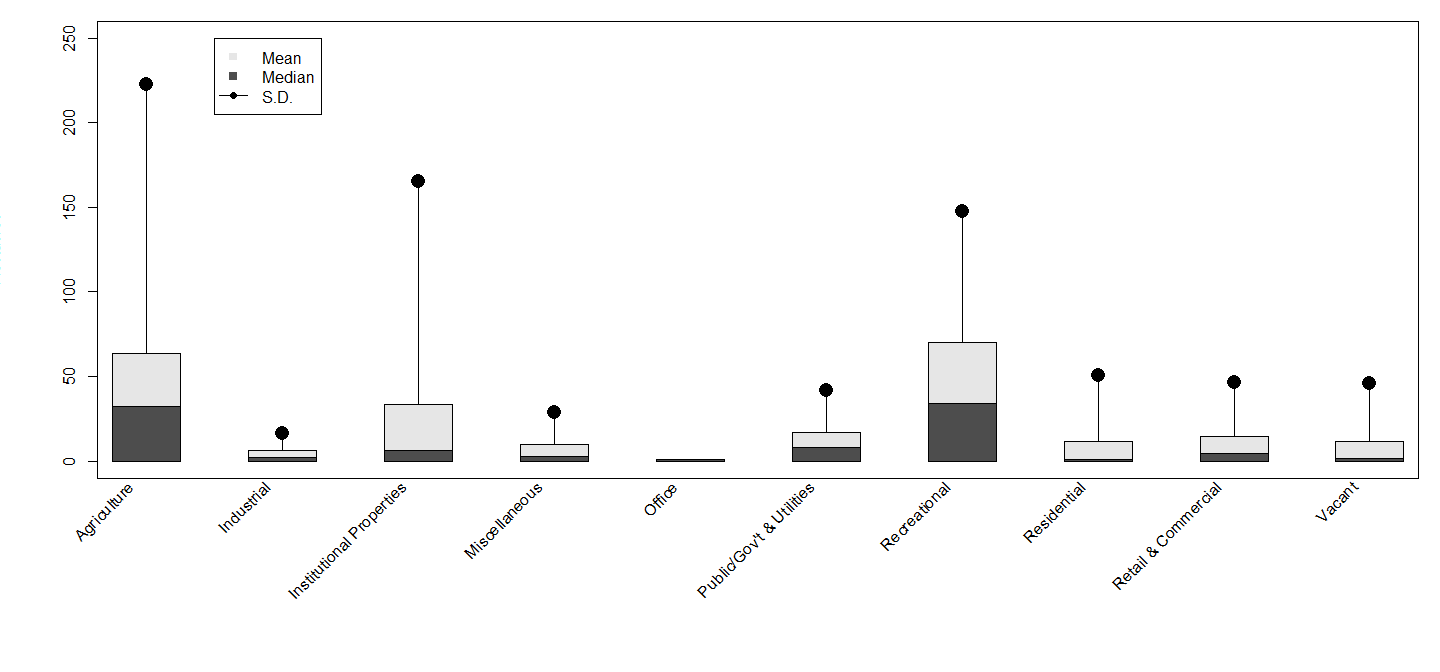

Supplement: Appendix S6 [file NIHMS1988779-supplement-Appendix_S6.tiff]

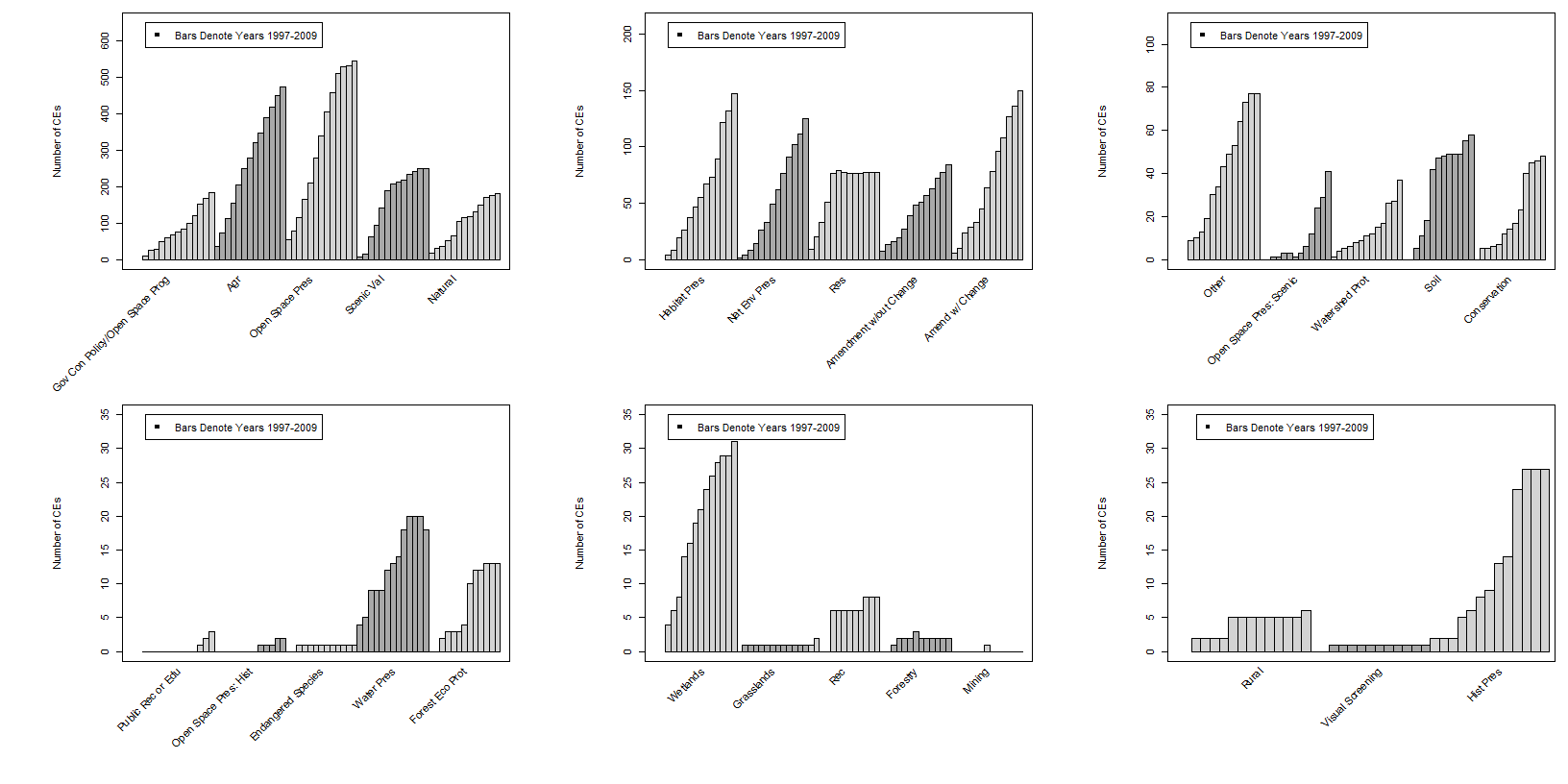

Supplement: Appendix S7 [file NIHMS1988779-supplement-Appendix_S7.tiff]
