# Supplementary material for: Unpacking conservation easements’ assessed land use designations and their implications for realizing biodiversity protection
Source: Conserv Sci Pract. Author manuscript; Available in PMC 2025 Jun 1. (PMC11675600; doi:10.1111/csp2.13130)
Supplement: Appendix S4 [file NIHMS1988779-supplement-Appendix_S4.docx]

**Appendix S4: Top 3 land use designations for each county’s parcels that have a CE placed at some point over our time frame and for parcels with a CE in a specific year + the number of CEs, parcel sizes on average + range, and the number of parcels with CEs over time, cumulative, in each county***

*Note: All parcels sizes are in hectares. Some of the counties do not have assessment information for some years, meaning that they have no land use codes for those year(s)—even if CEs were added. The turquoise indicates the highest mean CE parcel size for each county over the time period.

| **County** | | **Year** | | | | | | | | | | | | |
| --- | --- | --- | --- | --- | --- | --- | --- | --- | --- | --- | --- | --- | --- | --- |
|  |  | **1997** | **1998** | **1999** | **2000** | **2001** | **2002** | **2003** | **2004** | **2005** | **2006** | **2007** | **2008** | **2009** |
| **Albemarle** |  | -- | -- | Vacant Resid.  (285) | Vacant Resid. (379) | Vacant Resid.  (390) | Vacant Resid. (419) | Vacant Resid.  (428) | Vacant Resid.  (440) | Vacant Resid.  (465) | Vacant Resid.  (481) | Vacant Resid. (481) | Vacant Resid. (489) | Vacant Resid.  (507) |
|  | LU types |  |  | Single | Single | Single | Single | Single | Single | Single | Single | Single | Single | Single |
|  | for parcels |  |  | Family | Family | Family | Family | Family | Family | Family | Family | Family | Family | Family |
|  | with a CE | -- | -- | Resid. : | Resid. : | Resid. : | Resid. : | Resid. : | Resid. : | Resid. : | Resid. : | Resid. : | Resid. : | Resid. : |
|  | at some |  |  | dwelling | dwelling | dwelling | dwelling | dwelling | dwelling | dwelling | dwelling | dwelling | dwelling | dwelling |
|  | time during |  |  | (270) | (339) | (342) | (344) | (350) | (352) | (354) | (358) | (371) | (372) | (364) |
|  | our |  |  | Ag.: Misc. | Ag.: Misc. Farm; Ranch Support Buildings (3) | Ag.: Misc. | Ag.: Misc. Farm; Ranch Support Buildings (3) | Ag.: Misc. | Ag.: Misc. | Ag.: Misc. | Ag.: Misc. | Ag.: Misc. Farm; Ranch Support Buildings (5) | Ag.: Misc. Farm; Ranch Support Buildings (5) | Ag.: Misc. |
|  | timeframe |  |  | Farm; |  | Farm; |  | Farm; | Farm; | Farm; | Farm; |  |  | Farm; |
|  | (count) | -- | -- | Ranch  Support |  | Ranch  Support |  | Ranch  Support | Ranch  Support | Ranch  Support | Ranch  Support |  |  | Ranch  Support |
|  |  |  |  | Buildings |  | Buildings |  | Buildings | Buildings | Buildings | Buildings |  |  | Buildings |
|  |  |  |  | (3) |  | (3) |  | (5) | (5) | (5) | (5) |  |  | (10) |
|  |  | -- | -- | Vacant Resid. (24) | Vacant Resid. (92) | Vacant Resid.  (127) | Vacant Resid. (153) | Vacant Resid.  (189) | Vacant Resid.  (214) | Vacant Resid.  (333) | Vacant Resid.  (419) | Vacant Resid. (444) | Vacant Resid. (481) | Vacant Resid.  (519) |
|  |  |  |  | Single | Single | Single | Single | Single | Single | Single | Single | Single | Single | Single |
|  | LU types |  |  | Family | Family | Family | Family | Family | Family | Family | Family | Family | Family | Family |
|  | for those | -- | -- | Resid. : | Resid. : | Resid. : | Resid. : | Resid. : | Resid. : | Resid. : | Resid. : | Resid. : | Resid. : | Resid. : |
|  | parcels |  |  | dwelling | dwelling | dwelling | dwelling | dwelling | dwelling | dwelling | dwelling | dwelling | dwelling | dwelling |
|  | with CEs in |  |  | (24) | (75) | (94) | (126) | (169) | (203) | (252) | (290) | (347) | (354) | (376) |
|  | that year |  |  |  |  | Ag.: Misc. | Ag.: Misc. Farm; Ranch Support Buildings (1) | Ag.: Misc. | Ag.: Misc. | Ag.: Misc. | Ag.: Misc. | Ag.: Misc. Farm; Ranch Support Buildings (5) | Ag.: Misc. Farm; Ranch Support Buildings (5) | Ag.: Misc. |
|  | (count) |  |  |  |  | Farm; |  | Farm; | Farm; | Farm; | Farm; |  |  | Farm; |
|  |  | -- | -- | -- | -- | Ranch  Support |  | Ranch  Support | Ranch  Support | Ranch  Support | Ranch  Support |  |  | Ranch  Support |
|  |  |  |  |  |  | Buildings |  | Buildings | Buildings | Buildings | Buildings |  |  | Buildings |
|  |  |  |  |  |  | (1) |  | (1) | (3) | (5) | (5) |  |  | (5) |
|  | Total # of | 28 | 91 | 107 | 191 | 245 | 305 | 385 | 445 | 616 | 739 | 822 | 861 | 912 |
|  | CE parcels |  |  |  |  |  |  |  |  |  |  |  |  |  |
|  | # of CEs |  |  |  |  |  |  |  |  |  |  |  |  |  |
|  | added by | 16 | 21 | 10 | 34 | 21 | 26 | 29 | 29 | 44 | 44 | 41 | 15 | 31 |
|  | Year |  |  |  |  |  |  |  |  |  |  |  |  |  |
|  | Avg. CE | 56.66 | 35.54 | 35.52 | 36.05 | 35.30 | 33.44 | 34.61 | 35.91 | 35.17 | 33.31 | 33.56 | 33.87 | 33.49 |
|  | parcel size |  |  |  |  |  |  |  |  |  |  |  |  |  |
|  | Median CE | 28.82 | 12.11 | 13.63 | 14.78 | 12.72 | 14.78 | 15.53 | 15.70 | 15.70 | 15.39 | 15.52 | 15.70 | 15.74 |
|  | parcel size |  |  |  |  |  |  |  |  |  |  |  |  |  |
|  | Min CE | 0.65 | 0.08 | 0.08 | 0.05 | 0.05 | 0.05 | 0.05 | 0.05 | 0.05 | 0.05 | 0.05 | 0.05 | 0.05 |
|  | parcel size |  |  |  |  |  |  |  |  |  |  |  |  |  |

|  | Max CE parcel size | 308.56 | 308.56 | 308.56 | 310.55 | 358.76 | 358.76 | 358.76 | 358.76 | 380.68 | 380.68 | 380.68 | 380.68 | 380.68 |
| --- | --- | --- | --- | --- | --- | --- | --- | --- | --- | --- | --- | --- | --- | --- |
|  | St’d  deviation | 83.32 | 61.70 | 58.53 | 56.75 | 57.61 | 53.41 | 51.61 | 54.33 | 53.77 | 51.01 | 50.06 | 49.86 | 49.45 |
| **Boulder** |  | Ag.; | Ag.; | Ag.; | Ag.; Crops; field crops (irrigated and dry) (132) | Ag.; | Ag.; Crops; field crops (irrigated and dry) (152) | Ag.; | Ag.; | Ag.; | Ag.; | Ag.; Crops; field crops (irrigated and dry) (175) |  |  |
|  |  | Crops; | Crops; | Crops; |  | Crops; |  | Crops; | Crops; | Crops; | Crops; |  |  |  |
|  |  | field crops  (irrigated | field crops  (irrigated | field crops  (irrigated |  | field crops  (irrigated |  | field crops  (irrigated | field crops  (irrigated | field crops  (irrigated | field crops  (irrigated |  | -- | -- |
|  |  | and dry) | and dry) | and dry) |  | and dry) |  | and dry) | and dry) | and dry) | and dry) |  |  |  |
|  |  | (123) | (124) | (132) |  | (136) |  | (154) | (165) | (173) | (175) |  |  |  |
|  |  | Industrial; | Industrial; | Industrial; |  |  |  | Residential; SF 1-4;  single family dwelling (78) | Residential; SF 1-4;  single family dwelling (87) | Residential; SF 1-4;  single family dwelling (96) | Residential; SF 1-4;  single family dwelling (99) |  |  |  |
|  |  | Mining; | Mining; | Mining; |  |  |  |  |  |  |  | Public; Gov't |  |  |
|  | LU types | mineral | mineral | mineral |  |  |  |  |  |  |  | & Utilities; |  |  |
|  | for parcels | rights | rights | rights |  |  |  |  |  |  |  | County; |  |  |
|  | with a CE | (mining, | (mining, | (mining, | Misc. (64) | Misc. (70) | Misc. (69) |  |  |  |  | including | -- | -- |
|  | at some | timber, | timber, | timber, |  |  |  |  |  |  |  | land, |  |  |
|  | time during | etc.) and | etc.) and | etc.) and |  |  |  |  |  |  |  | buildings, |  |  |
|  | our | associated | associated | associated |  |  |  |  |  |  |  | etc. (123) |  |  |
|  | timeframe | land (59) | land (58) | land (57) |  |  |  |  |  |  |  |  |  |  |
|  | (count) |  | Residential  ; SF 1-4;  single family dwelling (46) |  | Industrial; | Residential; SF 1-4;  single family dwelling (59) |  |  |  |  |  |  |  |  |
|  |  |  |  |  | Mining; |  |  |  |  |  |  |  |  |  |
|  |  | Ag.; Misc |  |  | mineral |  | Residential; |  |  |  |  | Residential; |  |  |
|  |  | Ag; |  |  | rights |  | SF 1-4; |  |  |  |  | SF 1-4; |  |  |
|  |  | Mixed |  | Misc. (57) | (mining, |  | single family | Misc. (71) | Misc. (71) | Misc. (73) | Misc. (70) | single family | -- | -- |
|  |  | Agricultur |  |  | timber, etc.) |  | dwelling |  |  |  |  | dwelling |  |  |
|  |  | al (47) |  |  | and |  | (65) |  |  |  |  | (107) |  |  |
|  |  |  |  |  | associated |  |  |  |  |  |  |  |  |  |
|  |  |  |  |  | land (55) |  |  |  |  |  |  |  |  |  |
|  | LU types | Ag.; | Ag.;  Crops; field crops (irrigated and dry) (21) | Ag.; |  | Ag.; | Ag.; Crops; field crops (irrigated and dry) (134) | Ag.; | Ag.; | Ag.; | Ag.; | Ag.; Crops; field crops (irrigated and dry) (178) |  |  |
|  | for those | Crops; |  | Crops; | Ag.; Crops; | Crops; |  | Crops; | Crops; | Crops; | Crops; |  |  |  |
|  | parcels  with CEs in | field crops  (irrigated |  | field crops  (irrigated | field crops  (irrigated | field crops  (irrigated |  | field crops  (irrigated | field crops  (irrigated | field crops  (irrigated | field crops  (irrigated |  | -- | -- |
|  | that year | and dry) |  | and dry) | and dry) (81) | and dry) |  | and dry) | and dry) | and dry) | and dry) |  |  |  |
|  | (count) | (21) |  | (68) |  | (110) |  | (135) | (149) | (163) | (192) |  |  |  |
|  |  |  | Residential  ; SF 1-4;  single family dwelling (11) | Ag.;  Pasture; dry pasture (23) |  |  |  | Residential; SF 1-4;  single family dwelling (68) | Residential; SF 1-4;  single family dwelling (77) | Residential; SF 1-4;  single family dwelling (90) | Residential; SF 1-4;  single family dwelling (83) | Public; Gov't |  |  |
|  |  | Ag.; |  |  |  |  |  |  |  |  |  | & Utilities; |  |  |
|  |  | Pasture; |  |  |  |  |  |  |  |  |  | County; |  |  |
|  |  | dry |  |  | Misc. (49) | Misc. (55) | Misc. (53) |  |  |  |  | including | -- | -- |
|  |  | pasture |  |  |  |  |  |  |  |  |  | land, |  |  |
|  |  | (6) |  |  |  |  |  |  |  |  |  | buildings, |  |  |
|  |  |  |  |  |  |  |  |  |  |  |  | etc. (134) |  |  |
|  |  | Residentia |  | Residential; | Public; Gov't & Utilities; City; including land, buildings,  etc. (34) | Ag.; Misc.  Ag.;  Mixed Agricultura l (43) | Residential; SF 1-4;  single family dwelling (46) |  |  |  |  | Residential; SF 1-4;  single family dwelling (94) |  |  |
|  |  | l; SF 1-4; | Ag.; | SF 1-4; |  |  |  |  |  |  |  |  |  |  |
|  |  | single  family | Pasture;  dry pasture | single  family |  |  |  | Misc. (66) | Misc. (68) | Misc. (70) | Misc. (71) |  | -- | -- |
|  |  | dwelling | (10) | dwelling |  |  |  |  |  |  |  |  |  |  |
|  |  | (5) |  | (18) |  |  |  |  |  |  |  |  |  |  |

|  | Total # of CE parcels | 58 | 118 | 301 | 374 | 498 | 570 | 682 | 778 | 772 | 835 | 852 | 860 | 859 |
| --- | --- | --- | --- | --- | --- | --- | --- | --- | --- | --- | --- | --- | --- | --- |
|  | # of CEs added by  Year | 45 | 42 | 69 | 69 | 90 | 53 | 48 | 34 | 32 | 45 | 25 | 9 | -- |
|  | Avg. CE  parcel size | 37.08 | 31.79 | 23.83 | 23.32 | 22.25 | 20.82 | 21.61 | 21.77 | 21.92 | 20.98 | 21.25 | 21.23 | 21.26 |
|  | Median CE  parcel size | 14.10 | 13.45 | 11.61 | 10.57 | 9.23 | 8.07 | 7.86 | 7.28 | 7.64 | 7.82 | 7.91 | 7.98 | 8.00 |
|  | Min CE  parcel size | 0.16 | 0.00 | 0.00 | 0.00 | 0.00 | 0.00 | 0.00 | 0.00 | 0.00 | 0.00 | 0.00 | 0.00 | 0.00 |
|  | Max CE parcel size | 285.52 | 285.52 | 285.52 | 285.52 | 285.52 | 285.52 | 386.71 | 386.71 | 386.71 | 386.71 | 386.71 | 386.71 | 386.71 |
|  | St’d  deviation | 61.28 | 57.83 | 43.61 | 41.36 | 39.43 | 38.28 | 39.72 | 42.05 | 41.68 | 38.45 | 39.31 | 39.19 | 39.20 |
| **Charleston** | LU types for parcels with a CE at some time during our timeframe (count) | Ag. (127) | Ag. (128) | Residential; SF 1-4;  single family dwelling  (156) | Residential; SF 1-4;  single family dwelling (158) | Residential; SF 1-4;  single family dwelling  (162) | Residential; SF 1-4;  single family dwelling (217) | Residential; SF 1-4;  single family dwelling  (229) | Residential; SF 1-4;  single family dwelling  (318) | Residential; SF 1-4;  single family dwelling  (318) | Residential; SF 1-4;  single family dwelling  (364) | Residential; SF 1-4;  single family dwelling (352) | Residential; SF 1-4;  single family dwelling (355) | Residential; SF 1-4;  single family dwelling  (355) |
|  |  | Residentia l; SF 1-4;  single family dwelling (83) | Residential  ; SF 1-4;  single family dwelling (113) | Ag. (116) | Ag. (120) | Ag. (121) | Ag. (121) | Ag. (119) | Ag. (116) | Ag. (118) | Ag. (118) | Ag. (140) | Ag. (140) | Ag. (147) |
|  |  | Vacant; Vacant Residentia l (15) | Vacant; Misc Vacant; Undevelop  able (71) | Vacant; Misc Vacant; Undevelopa  ble (73) | Vacant; Misc Vacant;  Undevelopab le (76) | Vacant; Misc Vacant; Undevelopa  ble (75) | Vacant; Misc Vacant;  Undevelopab le (77) | Vacant; Misc Vacant; Undevelopa  ble (76) | Vacant; Misc Vacant; Undevelopa  ble (76) | Vacant; Misc Vacant; Undevelopa  ble (78) | Vacant; Misc Vacant; Undevelopa  ble (79) | Vacant; Misc Vacant;  Undevelopab le (79) | Vacant; Misc Vacant;  Undevelopab le (80) | Vacant; Misc Vacant; Undevelopa  ble (79) |
|  | LU types for those parcels with CEs in that year (count) | Ag. (8) | Vacant; Misc. Vacant; Undevelop able (65) | Residential; SF 1-4;  single family dwelling  (77) | Residential; SF 1-4;  single family dwelling (83) | Residential; SF 1-4;  single family dwelling  (94) | Residential; SF 1-4;  single family dwelling (212) | Residential; SF 1-4;  single family dwelling  (232) | Residential; SF 1-4;  single family dwelling  (412) | Residential; SF 1-4;  single family dwelling  (413) | Residential; SF 1-4;  single family dwelling  (511) | Residential; SF 1-4;  single family dwelling (522) | Residential; SF 1-4;  single family dwelling (536) | Residential; SF 1-4;  single family dwelling  (545) |
|  |  | Residentia l; SF 1-4;  single family dwelling  (8) | Residential  ; SF 1-4;  single family dwelling  (35) | Vacant; Misc. Vacant; Undevelopa ble (73) | Vacant; Misc. Vacant; Undevelopab le (74) | Vacant; Misc. Vacant; Undevelopa ble (75) | Vacant; Misc. Vacant; Undevelopab le (75) | Vacant; Misc. Vacant; Undevelopa ble (78) | Vacant; Misc. Vacant; Undevelopa ble (78) | Vacant; Misc. Vacant; Undevelopa ble (81) | Vacant; Misc. Vacant; Undevelopa ble (86) | Ag. (115) | Ag. (128) | Ag. (153) |
|  |  | Vacant; Vacant Retail & Comm’l  (7) | Ag. (15) | Ag. (32) | Ag. (30) | Ag. (36) | Residential; SF 1-4;  condominiu m (74) | Residential; SF 1-4;  condominiu m (76) | Residential; SF 1-4;  condominiu m (76) | Residential; SF 1-4;  condominiu m (76) | Residential; SF 1-4;  condominiu m (76) | Vacant; Misc. Vacant; Undevelopab  le (86) | Vacant; Misc. Vacant; Undevelopab  le (87) | Vacant; Misc. Vacant; Undevelopa  ble (144) |

|  | Total # of CE parcels | 917 | 887 | 1321 | 955 | 1348 | 1009 | 1038 | 1058 | 1066 | 1095 | 1146 | 1169 | 1256 |
| --- | --- | --- | --- | --- | --- | --- | --- | --- | --- | --- | --- | --- | --- | --- |
|  | # of CEs  added by Year | 15 | 12 | 21 | 9 | 13 | 21 | 22 | 13 | 11 | 13 | 33 | 11 | 21 |
|  | Avg. CE  parcel size | 1.91 | 2.77 | 4.55 | 6.06 | 5.54 | 12.07 | 10.01 | 10.11 | 10.44 | 11.10 | 16.24 | 15.65 | 16.09 |
|  | Median CE  parcel size | 0.14 | 0.14 | 0.14 | 0.15 | 0.14 | 0.16 | 0.17 | 0.18 | 0.18 | 0.19 | 0.21 | 0.22 | 0.29 |
|  | Min CE  parcel size | 0.00 | 0.00 | 0.00 | 0.00 | 0.00 | 0.00 | 0.00 | 0.00 | 0.00 | 0.00 | 0.00 | 0.00 | 0.00 |
|  | Max CE parcel size | 390.03 | 390.03 | 1280.52 | 1280.52 | 1280.52 | 1285.21 | 1285.21 | 1285.21 | 1285.21 | 1285.21 | 1285.21 | 1285.21 | 1285.21 |
|  | St’d  deviation | 18.94 | 21.15 | 42.55 | 49.04 | 45.41 | 87.52 | 65.91 | 65.24 | 65.63 | 66.76 | 84.87 | 76.64 | 76.37 |
| **Douglas** |  | Ag. (90) | Ag. (93) | Ag. (91) | Ag. (91) | Ag. (90) | Ag. (91) | Ag. (97) | Ag. (88) | Ag. (88) | Ag. (87) | Ag. (83) | Ag. (84) | -- |
|  | LU types for parcels with a CE at some time during our timeframe (count) | Residentia l; SF 1-4;  single family dwelling (4) | Residential  ; SF 1-4;  single family dwelling (4) | Residential; SF 1-4;  single family dwelling (4) | Residential; SF 1-4;  single family dwelling (4) | Ag.;  Pasture; irrigated or dry pasture with  residence(s) (5) | Ag.;  Pasture; irrigated or dry pasture with  residence(s) (5) | Ag.;  Pasture; irrigated or dry pasture with  residence(s) (5) | Residential; SF 1-4;  single family dwelling (5) | Residential; SF 1-4;  single family dwelling (5) | Misc.; Misc Riparian (10) | Misc.; Misc Riparian (12) | Misc.; Misc Riparian (12) | -- |
|  |  | Ag.;  Pasture; irrigated or dry pasture with residence(  s) (3) | Ag.;  Pasture; irrigated or dry pasture with residence(s  ) (3) | Ag.;  Pasture; irrigated or dry pasture with residence(s) (3) | Ag.;  Pasture; irrigated or dry pasture with residence(s) (4) | Residential; SF 1-4;  single family dwelling (4) | Residential; SF 1-4;  single family dwelling (4) | Residential; SF 1-4;  single family dwelling (4) | Ag.;  Pasture; irrigated or dry pasture with residence(s) (5) | Misc.; Misc Riparian (5) | Ag.;  Pasture; irrigated or dry pasture with residence(s) (4) | Residential; Lodging; resort motel (cabins, etc.) (5) | Residential; Lodging; resort motel (cabins, etc.) (5) | -- |
|  |  | -- | -- | -- | Ag. (6) | Ag. (16) | Ag. (70) | Ag. (95) | Ag. (87) | Ag. (90) | Ag. (88) | Ag. (89) | Ag. (89) | -- |
|  | LU types for those parcels with CEs in that year (count) | -- | -- | -- | -- | Residential; lodging; resort motel (1) | Ag.;  Pasture; irrigated or dry pasture with residences  (3) | Residential; SF 1-4;  single family dwelling (4) | Misc.; Misc Riparian (7) | Misc.; Misc Riparian (7) | Misc.; Misc Riparian (13) | Misc.; Misc Riparian (15) | Misc.; Misc Riparian (15) | -- |
|  |  | -- | -- | -- | -- | -- | Residential; Rural Res.; rural residential home site;  multi-family (3) | Ag.;  Pasture; irrigated or dry pasture with residence(s)  (4) | Residential; SF 1-4;  single family dwelling (5) | Residential; SF 1-4;  single family dwelling (5) | Ag.;  Pasture; irrigated or dry pasture with residence(s)  (4) | Residential; SF 1-4;  single family dwelling (5) | Residential; SF 1-4;  single family dwelling (6) | -- |
|  | Total # of  CE parcels | -- | -- | -- | 8 | 21 | 93 | 120 | 124 | 126 | 126 | 132 | 134 | 138 |

|  | # of CEs  added by Year | -- | -- | -- | 6 | 10 | 27 | 9 | 4 | 1 | 0 | 3 | 1 | 3 |
| --- | --- | --- | --- | --- | --- | --- | --- | --- | --- | --- | --- | --- | --- | --- |
|  | Avg. CE  parcel size | -- | -- | -- | 30.14 | 27.73 | 28.27 | 26.36 | 26.93 | 26.76 | 26.76 | 26.67 | 26.70 | 26.71 |
|  | Median CE  parcel | -- | -- | -- | 29.95 | 20.38 | 17.32 | 16.88 | 17.24 | 17.10 | 17.10 | 17.24 | 17.24 | 17.10 |
|  | Min CE  parcel size | -- | -- | -- | 3.03 | 3.03 | 2.12 | 2.12 | 2.12 | 2.12 | 2.12 | 2.12 | 2.12 | 2.12 |
|  | Max CE  parcel size | -- | -- | -- | 49.16 | 64.79 | 122.63 | 122.63 | 122.63 | 122.63 | 122.63 | 122.63 | 122.63 | 122.63 |
|  | St’d deviation | -- | -- | -- | 16.58 | 17.81 | 23.15 | 21.53 | 21.60 | 21.47 | 21.47 | 21.05 | 20.95 | 20.88 |
| **Greenville** | LU types for parcels with a CE at some time during our timeframe (count) | Vacant; Vacant Residentia  l (41) | Vacant; Vacant Residential  (41) | Vacant; Vacant Residential  (42) | Vacant; Vacant Residential  (43) | Vacant; Vacant Residential  (43) | Vacant; Vacant Residential  (47) | Vacant; Vacant Residential  (55) | Vacant; Vacant Residential  (59) | Vacant; Vacant Residential  (62) | Vacant; Vacant Residential  (70) | Vacant; Vacant Residential  (81) | Vacant; Vacant Residential  (165) | Vacant; Vacant Residential  (245) |
|  |  | Vacant; Vacant Ag (35) | Vacant; Vacant Ag (35) | Vacant; Vacant Ag (35) | Vacant; Vacant Ag (37) | Vacant; Vacant Ag (27) | Vacant; Vacant Ag (31) | Vacant; Vacant Ag (30) | Vacant; Vacant Ag (26) | Vacant; Vacant Ag (26) | Vacant; Vacant Ag (25) | Vacant; Vacant Ag (25) | Vacant; Vacant Ag (23) | Residential; SF 1-4;  single family  dwelling (25) |
|  |  | Vacant; Vacant Retail;  Comm (6) | Vacant; Vacant Retail; Comm (6) | Vacant; Vacant Retail; Comm (6) | Vacant; Vacant Retail; Comm (6) | Ag.;  Industrial Ag (12) | Ag.;  Industrial Ag (11) | Ag.;  Industrial Ag (11) | Ag.;  Industrial Ag (15) | Ag.;  Industrial Ag (15) | Ag.;  Industrial Ag (15) | Ag.;  Industrial Ag (16) | Ag.;  Industrial Ag (14) | Vacant; Vacant Ag (20) |
|  | LU types for those parcels with CEs in that year (count) | Vacant; Vacant Retail;  Comm (3) | Vacant; Vacant Retail; Comm (3) | Vacant; Vacant Retail; Comm (3) | Vacant; Vacant Retail; Comm (4) | Vacant; Vacant Ag (7) | Vacant; Vacant Ag (11) | Vacant; Vacant Residential (17) | Vacant; Vacant Residential (23) | Vacant; Vacant Residential (30) | Vacant; Vacant Residential (44) | Vacant; Vacant Residential (83) | Vacant; Vacant Residential (129) | Vacant; Vacant Residential (194) |
|  |  | Residentia l; SF 1-4;  single family dwelling  (1) | Residential  ; SF 1-4;  single family dwelling  (1) | Vacant; Vacant Ag (1) | Vacant; Vacant Ag (3) | Vacant; Vacant Retail; Comm (5) | Vacant; Vacant Residential (8) | Vacant; Vacant Ag (12) | Vacant; Vacant Ag (13) | Vacant; Vacant Ag (15) | Vacant; Vacant Ag (23) | Vacant; Vacant Ag (24) | Vacant; Vacant Ag (25) | Ag.;  Industrial Ag (26) |
|  |  | -- | Vacant; Vacant Residential  (1) | Vacant; Vacant Residential  (1) | Vacant; Vacant Residential  (2) | Vacant; Vacant Residential  (3) | Vacant; Vacant Retail;  Comm (6) | Ag.;  Industrial Ag (6) | Ag.;  Industrial Ag (7) | Ag.;  Industrial Ag (9) | Ag.;  Industrial Ag (17) | Ag.;  Industrial Ag (19) | Ag.;  Industrial Ag (18) | Vacant; Vacant Ag (24) |
|  | Total # of CE parcels | 7 | 8 | 9 | 17 | 31 | 52 | 59 | 133 | 152 | 185 | 368 | 305 | 319 |
|  | # of CEs added by  Year | 6 | 1 | 1 | 6 | 9 | 14 | 7 | 6 | 10 | 23 | 20 | 5 | 8 |

|  | Avg. CE parcel size | 41.10 | 36.18 | 52.95 | 39.82 | 29.43 | 18.14 | 196.38 | 88.70 | 81.88 | 75.69 | 39.36 | 82.68 | 47.07 |
| --- | --- | --- | --- | --- | --- | --- | --- | --- | --- | --- | --- | --- | --- | --- |
|  | Median CE  parcel size | 3.25 | 2.48 | 3.25 | 6.57 | 5.81 | 2.78 | 3.69 | 0.09 | 0.54 | 2.20 | 0.07 | 0.08 | 0.11 |
|  | Min CE  parcel size | 0.02 | 0.02 | 0.02 | 0.02 | 0.02 | 0.02 | 0.02 | 0.02 | 0.02 | 0.02 | 0.01 | 0.01 | 0.01 |
|  | Max CE parcel size | 228.87 | 228.87 | 228.87 | 228.87 | 228.87 | 228.87 | 7692.61 | 7692.61 | 7692.61 | 7692.61 | 7692.61 | 7692.61 | 7692.61 |
|  | St’d  deviation | 84.09 | 79.09 | 89.47 | 68.10 | 55.38 | 44.68 | 1057.88 | 707.92 | 662.28 | 600.35 | 428.01 | 660.98 | 459.52 |
| **Lebanon** | LU types for parcels with a CE at some time during our timeframe (count) | Agricultur e; Misc Ag; Agricultur al with buildings over 10  acres (96) | Agriculture  ; Misc Ag; Agricultura l with buildings over 10  acres (97) | Agriculture  ; Misc Ag; Agricultura l with buildings over 10  acres (97) | Agriculture; Misc Ag; Agricultural with buildings over 10 acres  (96) | Agriculture  ; Misc Ag; Agricultura l with buildings over 10  acres (97) | Agriculture; Misc Ag; Agricultural with buildings over 10 acres  (100) | Agriculture  ; Misc Ag; Agricultura l with buildings over 10  acres (100) | Agriculture  ; Misc Ag; Agricultura l with buildings over 10  acres (100) | Agriculture  ; Misc Ag; Agricultura l with buildings over 10  acres (100) | Agriculture  ; Misc Ag; Farm; Ranch Support Buildings (97) | Agriculture; Misc Ag; Farm; Ranch Support Buildings (100) | Agriculture; Misc Ag; Farm; Ranch Support Buildings (95) | Agriculture  ; Misc Ag; Farm; Ranch Support Buildings (102) |
|  |  | Miscellan eous (17) | Miscellane ous (17) | Miscellane ous (17) | Miscellaneou s (19) | Miscellane ous (20) | Miscellaneou s (20) | Miscellane ous (20) | Miscellane ous (20) | Miscellane ous (20) | Vacant; Misc Vacant; Vacant land  - 10+ acres  (15) | Vacant; Misc Vacant; Vacant land - 10+ acres  (16) | Vacant; Misc Vacant; Vacant land - 10+ acres  (16) | Vacant; Misc Vacant; Vacant land  - 10+ acres  (16) |
|  |  | Residentia l (9) | Residential (9) | Residential (9) | Residential (9) | Residential (10) | Residential (8) | Residential (8) | Residential (8) | Residential (8) | Agriculture  ; Crops; field crops (irrigated and dry) with residence(s)  (9) | Agriculture; Crops; field crops (irrigated and dry) with residence(s) (10) | Agriculture; Crops; field crops (irrigated and dry) with residence(s) (10) | Agriculture  ; Crops; field crops (irrigated and dry) with residence(s)  (10) |
|  | LU types for those parcels with CEs in that year (count) | Agricultur e; Misc Ag; Agricultur al with buildings over 10  acres (3) | Agriculture  ; Misc Ag; Agricultura l with buildings over 10  acres (6) | Agriculture  ; Misc Ag; Agricultura l with buildings over 10  acres (6) | Agriculture; Misc Ag; Agricultural with buildings over 10 acres  (18) | Agriculture  ; Misc Ag; Agricultura l with buildings over 10  acres (23) | Agriculture; Misc Ag; Agricultural with buildings over 10 acres  (30) | Agriculture  ; Misc Ag; Agricultura l with buildings over 10  acres (34) | Agriculture  ; Misc Ag; Agricultura l with buildings over 10  acres (46) | Agriculture  ; Misc Ag; Agricultura l with buildings over 10  acres (54) | Agriculture  ; Misc Ag; Farm; Ranch Support Buildings (72) | Agriculture; Misc Ag; Farm; Ranch Support Buildings (87) | Agriculture; Misc Ag; Farm; Ranch Support Buildings (92) | Agriculture  ; Misc Ag; Farm; Ranch Support Buildings (108) |
|  |  | -- | -- | Miscellane ous (1) | Miscellaneou s (6) | Miscellane ous (8) | Miscellaneou s (11) | Miscellane ous (12) | Miscellane ous (13) | Miscellane ous (16) | Vacant; Misc Vacant; Vacant land  - 10+ acres  (12) | Vacant; Misc Vacant; Vacant land - 10+ acres  (16) | Vacant; Misc Vacant; Vacant land - 10+ acres  (17) | Vacant; Misc Vacant; Vacant land  - 10+ acres  (17) |
|  |  | -- | -- | Miscellane ous; | Vacant (2) | Vacant (2) | Residential (2) | Residential (2) | Residential (3) | Residential (3) | Agriculture  ; Crops; field crops | Agriculture; Crops; field  crops | Agriculture; Crops; field  crops | Agriculture  ; Crops; field crops |

|  |  |  |  | Unknown |  |  |  |  |  |  | (irrigated | (irrigated | (irrigated | (irrigated |
| --- | --- | --- | --- | --- | --- | --- | --- | --- | --- | --- | --- | --- | --- | --- |
|  |  |  |  | (1) |  |  |  |  |  |  | and dry) | and dry) with | and dry) with | and dry) |
|  |  |  |  |  |  |  |  |  |  |  | with | residence(s) | residence(s) | with |
|  |  |  |  |  |  |  |  |  |  |  | residence(s) | (8) | (9) | residence(s) |
|  |  |  |  |  |  |  |  |  |  |  | (7) |  |  | (10) |
|  | Total # of | 3 | 6 | 12 | 28 | 35 | 47 | 53 | 68 | 81 | 105 | 124 | 144 | 153 |
|  | CE parcels |  |  |  |  |  |  |  |  |  |  |  |  |  |
|  | # of CEs |  |  |  |  |  |  |  |  |  |  |  |  |  |
|  | added by | 2 | 3 | 4 | 8 | 5 | 8 | 4 | 14 | 10 | 16 | 15 | 12 | 10 |
|  | Year |  |  |  |  |  |  |  |  |  |  |  |  |  |
|  | Avg. CE | 43.77 | 47.46 | 42.51 | 28.73 | 29.94 | 33.07 | 32.33 | 34.53 | 35.25 | 36.17 | 35.05 | 34.58 | 34.26 |
|  | parcel size |  |  |  |  |  |  |  |  |  |  |  |  |  |
|  | Median CE | 37.38 | 44.31 | 40.15 | 24.09 | 30.53 | 36.02 | 31.32 | 36.70 | 37.79 | 38.06 | 37.58 | 35.95 | 35.69 |
|  | parcel size |  |  |  |  |  |  |  |  |  |  |  |  |  |
|  | Min CE | 36.02 | 36.02 | 9.01 | 0.47 | 0.47 | 0.47 | 0.47 | 0.47 | 0.47 | 0.47 | 0.47 | 0.45 | 0.45 |
|  | parcel size |  |  |  |  |  |  |  |  |  |  |  |  |  |
|  | Max CE | 57.92 | 64.80 | 72.01 | 72.01 | 72.01 | 90.27 | 90.27 | 90.27 | 90.27 | 90.27 | 90.27 | 91.41 | 91.41 |
|  | parcel size |  |  |  |  |  |  |  |  |  |  |  |  |  |
|  | St’d | 12.27 | 11.68 | 16.68 | 18.84 | 19.45 | 20.91 | 20.39 | 19.54 | 19.56 | 19.45 | 19.77 | 20.63 | 20.37 |
|  | deviation |  |  |  |  |  |  |  |  |  |  |  |  |  |
| **Loudoun** |  |  |  |  |  | Vacant; | Vacant; | Vacant; | Vacant; | Vacant; | Vacant; | Vacant; | Vacant; | Vacant; |
|  |  | -- | -- | -- | -- | Vacant  Residential | Vacant  Residential | Vacant  Residential | Vacant  Residential | Vacant  Residential | Vacant  Residential | Vacant  Residential | Vacant  Residential | Vacant  Residential |
|  |  |  |  |  |  | (301) | (450) | (752) | (950) | (1200) | (1359) | (1479) | (1477) | (1579) |
|  | LU types |  |  |  |  | Residential; | Vacant; Misc Vacant; 10.0 Acres but <  35.0 Acres (144) | Vacant; | Vacant; | Vacant; | Vacant; | Vacant; Misc Vacant; 10.0 Acres but <  35.0 Acres (301) |  |  |
|  | for parcels with a CE at some  time during | -- | -- | -- | -- | SF 1-4;  single family  dwelling |  | Misc Vacant;  10.0 Acres  but < 35.0 | Misc Vacant;  10.0 Acres  but < 35.0 | Misc Vacant;  10.0 Acres  but < 35.0 | Misc Vacant;  10.0 Acres  but < 35.0 |  | Residential; Misc Res; HOA (364) | Residential; Misc Res; HOA (386) |
|  | our |  |  |  |  | (198) |  | Acres (191) | Acres (245) | Acres (265) | Acres (265) |  |  |  |
|  | timeframe |  |  |  |  | Vacant; | Residential; SF 1-4;  single family dwelling (113) | Residential; | Residential; | Residential; | Residential; | Residential; SF 1-4;  single family dwelling (158) | Vacant; Misc Vacant; 10.0 Acres but <  35.0 Acres (317) | Vacant; |
|  | (count) |  |  |  |  | Misc |  | SF 1-4; | SF 1-4; | SF 1-4; | SF 1-4; |  |  | Misc |
|  |  | -- | -- | -- | -- | Vacant;  10.0 Acres |  | single  family | single  family | single  family | single  family |  |  | Vacant;  10.0 Acres |
|  |  |  |  |  |  | but < 35.0 |  | dwelling | dwelling | dwelling | dwelling |  |  | but < 35.0 |
|  |  |  |  |  |  | Acres (125) |  | (117) | (139) | (162) | (188) |  |  | Acres (347) |
|  |  |  |  |  |  | Vacant; | Vacant; | Vacant; | Vacant; | Vacant; | Vacant; | Vacant; | Vacant; | Vacant; |
|  |  | -- | -- | -- | -- | Vacant  Residential | Vacant  Residential | Vacant  Residential | Vacant  Residential | Vacant  Residential | Vacant  Residential | Vacant  Residential | Vacant  Residential | Vacant  Residential |
|  | LU types |  |  |  |  | (295) | (427) | (700) | (820) | (1071) | (1240) | (1321) | (1316) | (1438) |
|  |  |  |  |  |  | Vacant; Misc Vacant;  10.0 Acres but < 35.0 Acres (119) |  | Vacant; Misc Vacant;  10.0 Acres but < 35.0 Acres (185) | Vacant; Misc Vacant;  10.0 Acres but < 35.0 Acres (239) | Vacant; Misc Vacant;  10.0 Acres but < 35.0 Acres (266) | Vacant; Misc Vacant;  10.0 Acres but < 35.0 Acres (266) |  |  |  |
|  | for those |  |  |  |  |  | Vacant; Misc |  |  |  |  | Vacant; Misc |  |  |
|  | parcels |  |  |  |  |  | Vacant; 10.0 |  |  |  |  | Vacant; 10.0 | Residential; | Residential; |
|  | with CEs in | -- | -- | -- | -- |  | Acres but < |  |  |  |  | Acres but < | Misc Res; | Misc Res; |
|  | that year |  |  |  |  |  | 35.0 Acres |  |  |  |  | 35.0 Acres | HOA (334) | HOA (355) |
|  | (count) |  |  |  |  |  | (140) |  |  |  |  | (298) |  |  |
|  |  |  |  |  |  | Residential; | Residential; | Residential; | Residential; | Residential; | Residential; | Residential; | Vacant; Misc | Vacant; |
|  |  | -- | -- | -- | -- | SF 1-4; | SF 1-4; | SF 1-4; | SF 1-4; | SF 1-4; | SF 1-4; | SF 1-4; | Vacant; 10.0 | Misc |
|  |  |  |  |  |  | single | single family | single | single | single | single | single family | Acres but < | Vacant; |

|  |  |  |  |  |  | family  dwelling (92) | dwelling (41) | family  dwelling (54) | family  dwelling (86) | family  dwelling (119) | family  dwelling (170) | dwelling (149) | 35.0 Acres (315) | 10.0 Acres  but < 35.0  Acres (345) |
| --- | --- | --- | --- | --- | --- | --- | --- | --- | --- | --- | --- | --- | --- | --- |
|  | Total # of CE parcels | 103 | 186 | 337 | 509 | 722 | 1212 | 1513 | 1848 | 2106 | 2340 | 2418 | 2472 | 2532 |
|  | # of CEs  added by Year | 21 | 16 | 22 | 21 | 44 | 76 | 40 | 48 | 35 | 60 | 27 | 24 | 19 |
|  | Avg. CE  parcel size | 10.49 | 12.29 | 8.62 | 7.18 | 7.62 | 6.75 | 6.29 | 6.10 | 6.43 | 6.69 | 6.92 | 7.07 | 7.43 |
|  | Median CE  parcel size | 6.01 | 7.78 | 4.35 | 1.85 | 1.23 | 0.80 | 0.63 | 0.55 | 0.54 | 0.54 | 0.55 | 0.55 | 0.56 |
|  | Min CE  parcel size | 0.02 | 0.02 | 0.02 | 0.02 | 0.01 | 0.01 | 0.01 | 0.00 | 0.00 | 0.00 | 0.00 | 0.00 | 0.00 |
|  | Max CE  parcel size | 93.84 | 109.50 | 109.50 | 109.50 | 266.56 | 266.56 | 266.56 | 266.56 | 369.07 | 369.07 | 369.07 | 369.07 | 369.07 |
|  | St’d deviation | 15.31 | 17.66 | 14.42 | 13.26 | 17.90 | 15.79 | 15.37 | 15.55 | 18.07 | 18.52 | 18.85 | 19.09 | 20.03 |
| **Mesa** | LU types for parcels with a CE at some time during our timeframe (count) | Ag.;  Pasture; dry  pasture (84) | Ag.;  Pasture; dry pasture (86) | Ag.;  Pasture; dry pasture (92) | Ag.;  Pasture; dry pasture (96) | Ag.;  Pasture; dry pasture (105) | Ag.;  Pasture; dry pasture (106) | Ag.;  Pasture; dry pasture (110) | Ag.;  Pasture; dry pasture (116) | Ag.;  Pasture; dry pasture (137) | Ag.;  Pasture; dry pasture (135) | Ag.;  Pasture; dry pasture (144) | Ag.;  Pasture; dry pasture (147) | -- |
|  |  | Ag.; Misc Ag; Mixed  Agricultur al (66) | Ag.; Misc Ag; Mixed Agricultura l (66) | Ag.; Misc Ag; Mixed Agricultura l (70) | Ag.; Misc Ag; Mixed Agricultural (75) | Ag.; Misc Ag; Mixed Agricultura l (77) | Ag.; Misc Ag; Mixed Agricultural (81) | Ag.; Misc Ag; Mixed Agricultura l (88) | Ag.; Misc Ag; Mixed Agricultura l (93) | Ag.; Misc Ag; Mixed Agricultura l (95) | Ag.; Misc Ag; Mixed Agricultura l (106) | Ag.; Misc Ag; Mixed Agricultural (107) | Ag.; Misc Ag; Mixed Agricultural (108) | -- |
|  |  | Ag.;  Pasture; irrigated or dry pasture  (28) | Ag.;  Pasture; irrigated or dry pasture (28) | Ag.;  Pasture; irrigated or dry pasture (27) | Ag.;  Pasture; irrigated or dry pasture (33) | Ag.;  Pasture; irrigated or dry pasture (27) | Ag.;  Pasture; irrigated or dry pasture (29) | Ag.;  Pasture; irrigated or dry pasture (32) | Ag.;  Pasture; irrigated or dry pasture (49) | Ag.;  Pasture; irrigated or dry pasture (45) | Ag.;  Pasture; irrigated or dry pasture (47) | Ag.;  Pasture; irrigated or dry pasture (49) | Ag.;  Pasture; irrigated or dry pasture (49) | -- |
|  |  | Ag.;  Pasture; dry pasture  (5) | Ag.;  Pasture; dry pasture (7) | Ag.;  Pasture; dry pasture (24) | Ag.;  Pasture; dry pasture (32) | Ag.;  Pasture; dry pasture (39) | Ag.;  Pasture; dry pasture (51) | Ag.;  Pasture; dry pasture (54) | Ag.;  Pasture; dry pasture (63) | Ag.;  Pasture; dry pasture (80) | Ag.;  Pasture; dry pasture (103) | Ag.;  Pasture; dry pasture (144) | Ag.;  Pasture; dry pasture (154) | -- |
|  | LU types for those parcels with CEs in that year (count) | Ag.; Misc Ag; Mixed  Agricultur al (1) | Ag.; Misc Ag; Mixed Agricultura l (2) | Ag.; Misc Ag; Mixed Agricultura l (15) | Ag.; Misc Ag; Mixed Agricultural (21) | Ag.; Misc Ag; Mixed Agricultura l (36) | Ag.; Misc Ag; Mixed Agricultural (47) | Ag.; Misc Ag; Mixed Agricultura l (45) | Ag.; Misc Ag; Mixed Agricultura l (54) | Ag.; Misc Ag; Mixed Agricultura l (63) | Ag.; Misc Ag; Mixed Agricultura l (82) | Ag.; Misc Ag; Mixed Agricultural (111) | Ag.; Misc Ag; Mixed Agricultural (107) | -- |
|  |  | Public Gov’t & Utilities; County; Admin.  (1) | Ag.;  Pasture; irrigated or dry pasture (1) | Ag.;  Pasture; irrigated or dry pasture (5) | Ag.;  Pasture; irrigated or dry pasture (7) | Ag.;  Pasture; irrigated or dry pasture (21) | Ag.;  Pasture; irrigated or dry pasture (21) | Ag.;  Pasture; irrigated or dry pasture (25) | Ag.;  Pasture; irrigated or dry pasture (26) | Ag.;  Pasture; irrigated or dry pasture (22) | Ag.;  Pasture; irrigated or dry pasture (23) | Ag.;  Pasture; irrigated or dry pasture (57) | Ag.;  Pasture; irrigated or dry pasture (45) | -- |

|  | Total # of CE parcels | 7 | 23 | 79 | 97 | 152 | 164 | 163 | 181 | 208 | 250 | 356 | 349 | 385 |
| --- | --- | --- | --- | --- | --- | --- | --- | --- | --- | --- | --- | --- | --- | --- |
|  | # of CEs  added by Year | 4 | 6 | 15 | 11 | 11 | 11 | 11 | 11 | 17 | 23 | 27 | 18 | 19 |
|  | Avg. CE  parcel size | 142.62 | 62.23 | 63.16 | 62.59 | 54.18 | 120.78 | 126.43 | 114.24 | 112.69 | 104.68 | 87.40 | 86.60 | 86.12 |
|  | Median CE  parcel size | 65.33 | 25.27 | 32.43 | 29.86 | 22.83 | 32.05 | 26.43 | 25.27 | 24.11 | 22.16 | 22.16 | 20.86 | 20.80 |
|  | Min CE  parcel size | 16.47 | 2.30 | 2.02 | 0.85 | 0.50 | 0.50 | 0.50 | 0.50 | 0.50 | 0.50 | 0.50 | 0.50 | 0.17 |
|  | Max CE parcel size | 475.28 | 475.28 | 550.09 | 550.09 | 550.09 | 3615.49 | 3615.49 | 3615.49 | 3615.49 | 3615.49 | 3615.49 | 3615.49 | 3615.49 |
|  | St’d  deviation | 164.05 | 104.03 | 100.09 | 105.17 | 90.81 | 434.63 | 437.71 | 415.53 | 407.20 | 378.42 | 320.81 | 322.21 | 312.63 |
| **Sacramento** | LU types for parcels with a CE at some time during our timeframe (count) | Ag.;  Pasture; dry pasture  (66) | Ag.;  Pasture; dry pasture (67) | Ag.;  Pasture; dry pasture (68) | Ag.;  Pasture; dry pasture (70) | Ag.;  Pasture; dry pasture (70) | Ag.;  Pasture; dry pasture (70) | Ag.;  Pasture; dry pasture (69) | Ag.;  Pasture; dry pasture (69) | Ag.;  Pasture; dry pasture (73) | Ag.;  Pasture; dry pasture (75) | Ag.;  Pasture; dry pasture (77) | Ag.;  Pasture; dry pasture (77) | -- |
|  |  | Ag.;  Pasture; irrigated or dry pasture  (17) | Ag.;  Pasture; irrigated or dry pasture (16) | Ag.;  Pasture; irrigated or dry pasture (15) | Ag.;  Pasture; irrigated or dry pasture (15) | Ag.;  Pasture; irrigated or dry pasture (16) | Ag.;  Pasture; irrigated or dry pasture (17) | Ag.;  Pasture; irrigated or dry pasture (19) | Ag.;  Pasture; irrigated or dry pasture (20) | Ag.;  Pasture; irrigated or dry pasture (22) | Ag.;  Pasture; irrigated or dry pasture (22) | Ag.;  Pasture; irrigated or dry pasture (22) | Ag.;  Pasture; irrigated or dry pasture (22) | -- |
|  |  | Ag.;  Crops; field crops (irrigated  and dry) (4) | Ag.;  Crops; field crops (irrigated  and dry) (4) | Ag.;  Crops; field crops (irrigated  and dry) (4) | Ag.; Crops; field crops (irrigated and dry) (4) | Ag.;  Crops; field crops (irrigated  and dry) (4) | Ag.; Crops; field crops (irrigated and dry) (4) | Vacant Resid.: Undevelope d land (5) | Vacant Resid.: Undevelope d land (6) | Vacant Resid.: Undevelope d land (8) | Residential; Misc Res; Common Area (condo;  PUD) (6) | Residential; Misc Res; Common Area (condo; PUD) (6) | Residential; Misc Res; Common Area (condo; PUD) (6) | -- |
|  |  | Ag.;  Pasture; dry  pasture (13) | Ag.;  Pasture; dry pasture (15) | Ag.;  Pasture; dry pasture (49) | Ag.;  Pasture; dry pasture (51) | Ag.;  Pasture; dry pasture (75) | Ag.;  Pasture; dry pasture (83) | Ag.;  Pasture; dry pasture (82) | Ag.;  Pasture; dry pasture (82) | Ag.;  Pasture; dry pasture (87) | Ag.;  Pasture; dry pasture (92) | Ag.;  Pasture; dry pasture (102) | Ag.;  Pasture; dry pasture (101) | -- |
|  | LU types for those parcels with CEs in that year (count) | Ag.;  Pasture; irrigated or dry pasture  (3) | Ag.;  Pasture; irrigated or dry pasture (4) | Ag.;  Pasture; irrigated or dry pasture (6) | Ag.;  Pasture; irrigated or dry pasture (16) | Ag.;  Pasture; irrigated or dry pasture (14) | Ag.;  Pasture; irrigated or dry pasture (16) | Ag.;  Pasture; irrigated or dry pasture (20) | Ag.;  Pasture; irrigated or dry pasture (22) | Ag.;  Pasture; irrigated or dry pasture (23) | Ag.;  Pasture; irrigated or dry pasture (23) | Ag.;  Pasture; irrigated or dry pasture (23) | Ag.;  Pasture; irrigated or dry pasture (23) | -- |
|  |  | Ag.;  Patures; field crop and dry pasture (1) | Ag.;  Patures; field crop and dry pasture (2) | Public Gov't & Utilities; State; state property including  buildings, | Ag.; Crops; field crops (irrigated and dry) (2) | Ag.;  Crops; field crops (irrigated and dry) (4) | Ag.; Crops; field crops (irrigated and dry) (4) | Vacant Resid.: Undevelope d land (5) | Vacant Resid.: Undevelope d land (6) | Vacant Resid.: Undevelope d land (15) | Residential; Misc Res; Common Area (condo; PUD) (6) | Residential; Misc Res; Common Area (condo; PUD) (6) | Residential; Misc Res; Common Area (condo; PUD) (6) | -- |

|  |  | lands, etc. (2) | | | | | | | | | | | | |
| --- | --- | --- | --- | --- | --- | --- | --- | --- | --- | --- | --- | --- | --- | --- |
|  | Total # of  CE parcels | 19 | 34 | 80 | 95 | 121 | 142 | 149 | 159 | 178 | 187 | 209 | 211 | 215 |
|  | # of CEs added by  Year | 5 | 4 | 4 | 5 | 6 | 6 | 3 | 6 | 5 | 10 | 10 | 4 | 2 |
|  | Avg. CE  parcel size | 77.23 | 64.51 | 119.45 | 106.56 | 124.89 | 112.66 | 109.54 | 104.05 | 93.17 | 97.93 | 99.57 | 99.91 | 99.49 |
|  | Median CE  parcel size | 37.26 | 38.47 | 70.56 | 62.45 | 81.61 | 64.04 | 62.45 | 60.31 | 43.39 | 48.61 | 57.74 | 57.74 | 57.74 |
|  | Min CE parcel size | 1.11 | 1.11 | 0.05 | 0.05 | 0.05 | 0.05 | 0.05 | 0.03 | 0.03 | 0.03 | 0.03 | 0.03 | 0.03 |
|  | Max CE  parcel size | 209.73 | 209.73 | 268.18 | 268.18 | 596.02 | 596.02 | 596.02 | 596.02 | 596.02 | 596.02 | 596.02 | 596.02 | 596.02 |
|  | St’d  deviation | 76.17 | 64.25 | 101.14 | 97.89 | 109.02 | 107.94 | 106.38 | 105.26 | 104.35 | 103.96 | 103.05 | 103.42 | 102.89 |
| **Sonoma** |  | -- | -- | -- | -- | -- | Ag.;  Pasture; irrigated or dry pasture  (85) | Ag.;  Pasture; irrigated or dry pasture  (85) | Ag.;  Pasture; irrigated or dry pasture  (87) | Ag.;  Pasture; irrigated or dry pasture  (87) | Ag.;  Pasture; irrigated or dry pasture  (95) | Ag.;  Pasture; irrigated or dry pasture  (95) | Ag.;  Pasture; irrigated or dry pasture  (95) | -- |
|  | LU types for parcels with a CE at some time during our timeframe (count) | -- | -- | -- | -- | -- | Vacant Resid.: Undeveloped land (32) | Vacant Resid.: Undevelope d land (32) | Vacant Resid.: Undevelope d land (31) | Vacant Resid.: Undevelope d land (31) | Ag.;  Timber; Forest ; timber preserve zone (different  lists) (33) | Ag.;  Timber; Forest ; timber preserve zone (different  lists) (34) | Ag.;  Timber; Forest ; timber preserve zone (different  lists) (34) | -- |
|  |  | -- | -- | -- | -- | -- | Ag.;  Timber; Forest ; timber preserve zone (different  lists) (28) | Ag.;  Timber; Forest ; timber preserve zone (different  lists) (28) | Ag.;  Timber; Forest ; timber preserve zone (different  lists) (30) | Ag.;  Timber; Forest ; timber preserve zone (different  lists) (30) | Vacant Resid.: Undevelope d land (33) | Vacant Resid.: Undeveloped land (33) | Vacant Resid.: Undeveloped land (33) | -- |
|  | LU types for those parcels with CEs in  that year (count) | -- | -- | -- | -- | -- | Ag.;  Pasture; irrigated or dry pasture (61) | Ag.;  Pasture; irrigated or dry pasture (64) | Ag.;  Crops; field crops (irrigated and dry) with manufactur  ed home(s) (69) | Ag.;  Pasture; irrigated or dry pasture (70) | Ag.;  Pasture; irrigated or dry pasture (80) | Ag.;  Pasture; irrigated or dry pasture (88) | -- | -- |
|  |  | -- | -- | -- | -- | -- | Residential;  Rural Res; rural | Residential;  Rural Res; rural | Ag.;  Timber; Forest ; | Ag.;  Timber; Forest; | Ag.;  Timber; Forest; | Ag.;  Timber; Forest; | -- | -- |

|  |  |  |  |  |  |  | residential | residential | timber | hardwoods | hardwoods | hardwoods |  |  |
| --- | --- | --- | --- | --- | --- | --- | --- | --- | --- | --- | --- | --- | --- | --- |
|  |  |  |  |  |  |  | home site | home site | preserve | and | and | and |  |  |
|  |  |  |  |  |  |  | (14) | (17) | zone | chaparral | chaparral | chaparral |  |  |
|  |  |  |  |  |  |  |  |  | (different | with or | with or | with or |  |  |
|  |  |  |  |  |  |  |  |  | lists) (20) | without | without | without |  |  |
|  |  |  |  |  |  |  |  |  |  | residence | residence | residence or |  |  |
|  |  |  |  |  |  |  |  |  |  | or | or | manufacture |  |  |
|  |  |  |  |  |  |  |  |  |  | manufactur | manufactur | d home (25) |  |  |
|  |  |  |  |  |  |  |  |  |  | ed home | ed home |  |  |  |
|  |  |  |  |  |  |  |  |  |  | (20) | (25) |  |  |  |
|  |  |  |  |  |  |  |  |  | Residential; | Residential; | Residential; | Residential; |  |  |
|  |  |  |  |  |  |  | Vacant | Vacant | Rural Res; | Rural Res; | Rural Res; | Rural Res; |  |  |
|  |  | -- | -- | -- | -- | -- | Resid.:  Undeveloped | Resid.:  Undevelope | rural  residential | rural  residential | rural  residential | rural  residential | -- | -- |
|  |  |  |  |  |  |  | land (10) | d land (15) | home site | home site | home site | home site |  |  |
|  |  |  |  |  |  |  |  |  | (19) | (19) | (26) | (22) |  |  |
|  | Total # of | 21 | 37 | 52 | 89 | 135 | 217 | 280 | 307 | 350 | 371 | 396 | 403 | 462 |
|  | CE parcels |  |  |  |  |  |  |  |  |  |  |  |  |  |
|  | # of CEs |  |  |  |  |  |  |  |  |  |  |  |  |  |
|  | added by | 6 | 7 | 7 | 9 | 4 | 20 | 14 | 14 | 14 | 9 | 8 | 5 | 8 |
|  | Year |  |  |  |  |  |  |  |  |  |  |  |  |  |
|  | Avg. CE | 35.40 | 38.32 | 36.49 | 50.06 | 69.58 | 56.38 | 55.97 | 55.16 | 62.66 | 59.88 | 60.14 | 59.37 | 57.86 |
|  | parcel size |  |  |  |  |  |  |  |  |  |  |  |  |  |
|  | Median CE | 16.68 | 15.97 | 14.30 | 24.60 | 29.06 | 20.27 | 20.66 | 20.78 | 23.33 | 21.51 | 21.60 | 21.51 | 20.75 |
|  | parcel size |  |  |  |  |  |  |  |  |  |  |  |  |  |
|  | Min CE | 0.28 | 0.28 | 0.13 | 0.09 | 0.09 | 0.04 | 0.04 | 0.04 | 0.04 | 0.04 | 0.04 | 0.04 | 0.04 |
|  | parcel size |  |  |  |  |  |  |  |  |  |  |  |  |  |
|  | Max CE | 167.48 | 199.95 | 227.83 | 577.66 | 577.66 | 577.66 | 577.66 | 577.66 | 577.66 | 577.66 | 577.66 | 577.66 | 577.66 |
|  | parcel size |  |  |  |  |  |  |  |  |  |  |  |  |  |
|  | St’d | 44.17 | 53.26 | 55.65 | 79.18 | 105.35 | 91.28 | 87.62 | 84.98 | 95.50 | 93.60 | 93.17 | 92.57 | 88.88 |
|  | deviation |  |  |  |  |  |  |  |  |  |  |  |  |  |
| **Washington** |  | Ag. (15) | Ag. (51) | Ag. (138) | Residential; SF 1-4 (155) | Residential; SF 1-4  (144) | Residential (186) | Residential (226) | Residential (260) | Residential (281) | Residential (295) | Residential (304) | Residential (341) | Residential (318) |
|  | LU types  for parcels with a CE | Residentia l (9) | Residential (9) | Residential; SF 1-4  (37) | Ag. (66) | Residential (126) | Residential; SF 1-4 (154) | Residential; SF 1-4  (142) | Residential; SF 1-4  (128) | Residential; SF 1-4  (120) | Residential; SF 1-4  (120) | Residential; SF 1-4 (140) | Residential; SF 1-4 (145) | Residential; SF 1-4  (156) |
|  | at some | Institution | Institutiona |  |  |  |  |  |  |  |  |  |  |  |
|  | time during | al | l |  |  |  |  |  |  |  |  |  |  |  |
|  | our  timeframe (count) | Properties  ; Misc Institution  al ; | Properties; Misc Institutiona  l ; | Residential (9) | Residential (45) | Ag. (98) | Ag. (32) | Ag. (39) | Ag. (34) | Ag. (29) | Misc.; Exempt (35) | Misc.; Exempt (48) | Misc.; Exempt (62) | Misc.; Exempt (74) |
|  |  | Charitable | Charitable |  |  |  |  |  |  |  |  |  |  |  |
|  |  | (7) | (7) |  |  |  |  |  |  |  |  |  |  |  |
|  | LU types for those  parcels | -- | Ag. (38) | Ag. (117) | Residential; SF 1-4 ; SF  1 – 5 (184) | Residential; SF 1-4 ; SF  1 – 5 (146) | Residential (168) | Residential (204) | Residential (232) | Residential (252) | Residential (252) | Residential (302) | Residential (340) | Residential (322) |

|  | with CEs in |  | Recreation |  |  |  |  |  |  |  |  |  |  |  |
| --- | --- | --- | --- | --- | --- | --- | --- | --- | --- | --- | --- | --- | --- | --- |
|  | that year (count) | -- | al; Misc Recreation  al; Private | Residential; SF 1-4 ; SF  1 – 5 (36) | Residential (56) | Residential (145) | Residential; SF 1-4 ; SF  1 – 5 (131) | Residential; SF 1-4 ; SF  1 – 5 (131) | Residential; SF 1-4 ; SF  1 – 5 (125) | Residential; SF 1-4 ; SF  1 – 5 (114) | Residential; SF 1-4 ; SF  1 – 5 (114) | Residential; SF 1-4 ; SF  1 – 5 (140) | Residential; SF 1-4 ; SF  1 – 5 (144) | Residential; SF 1-4 ; SF  1 – 5 (155) |
|  |  |  | Rec (1) |  |  |  |  |  |  |  |  |  |  |  |
|  |  |  | Ag.; |  |  |  |  |  |  |  |  |  |  |  |
|  |  |  | Timber; |  |  |  |  |  |  |  |  |  |  |  |
|  |  |  | Forest; | Residential; |  |  |  |  |  |  | Public |  |  |  |
|  |  |  | timber land | Rural Res; | Residential; |  |  |  |  |  | Gov’t & |  |  |  |
|  |  |  | with or | rural | SF 1-4; |  |  |  |  |  | Utilities; |  |  |  |
|  |  | -- | without | residential | single family | Ag. (55) | Ag. (26) | Ag. (34) | Ag. (25) | Ag. (20) | State; state | Ag. (23) | Ag. (25) | Ag. (24) |
|  |  |  | residence | home site; | dwelling |  |  |  |  |  | possessory |  |  |  |
|  |  |  | or | multi- | (11) |  |  |  |  |  | interest |  |  |  |
|  |  |  | manufactur | family (3) |  |  |  |  |  |  | (35) |  |  |  |
|  |  |  | ed home |  |  |  |  |  |  |  |  |  |  |  |
|  |  |  | (1) |  |  |  |  |  |  |  |  |  |  |  |
|  | Total # of CE parcels | 21 | 150 | 208 | 374 | 406 | 424 | 441 | 465 | 510 | 559 | 640 | 655 | 668 |
|  | # of CEs |  |  |  |  |  |  |  |  |  |  |  |  |  |
|  | added by | 7 | 22 | 48 | 35 | 34 | 39 | 15 | 8 | 11 | 17 | 11 | 3 | 5 |
|  | Year |  |  |  |  |  |  |  |  |  |  |  |  |  |
|  | Avg. CE | 4.79 | 2.33 | 2.33 | 1.68 | 1.71 | 2.38 | 2.44 | 2.44 | 2.64 | 2.82 | 2.77 | 2.76 | 2.95 |
|  | parcel size |  |  |  |  |  |  |  |  |  |  |  |  |  |
|  | Median CE | 1.30 | 0.34 | 0.25 | 0.15 | 0.15 | 0.18 | 0.18 | 0.21 | 0.28 | 0.37 | 0.39 | 0.37 | 0.39 |
|  | parcel size |  |  |  |  |  |  |  |  |  |  |  |  |  |
|  | Min CE | 0.13 | 0.05 | 0.05 | 0.01 | 0.01 | 0.01 | 0.01 | 0.01 | 0.01 | 0.01 | 0.01 | 0.01 | 0.01 |
|  | parcel size |  |  |  |  |  |  |  |  |  |  |  |  |  |
|  | Max CE | 47.17 | 47.17 | 47.17 | 47.17 | 47.17 | 47.17 | 47.17 | 47.17 | 47.17 | 47.17 | 47.17 | 47.17 | 61.39 |
|  | parcel size |  |  |  |  |  |  |  |  |  |  |  |  |  |
|  | St’d | 10.80 | 5.70 | 5.59 | 4.60 | 4.63 | 5.46 | 5.73 | 5.27 | 5.43 | 5.40 | 5.49 | 5.61 | 6.21 |
|  | deviation |  |  |  |  |  |  |  |  |  |  |  |  |  |
| **York** | LU types | Agricultur e; Crops  (481) | Agriculture  ; Crops (354) | Agriculture  ; Crops (341) | Agriculture; Crops (335) | Agriculture  ; Crops (335) | Agriculture; Crops (335) | Agriculture  ; Crops (336) | Agriculture  ; Crops (330) | Agriculture  ; Crops (330) | Agriculture  ; Crops (326) | Agriculture; Crops (319) | Agriculture; Crops (318) | Agriculture  ; Crops (317) |
|  |  | Residentia l; SF 1-4;  single family dwelling (196) | Residential  ; SF 1-4;  single family dwelling (200) | Residential; SF 1-4;  single family dwelling (209) |  | Residential; SF 1-4;  single family dwelling (226) |  | Residential; SF 1-4;  single family dwelling (240) | Residential; SF 1-4;  single family dwelling (253) | Residential; SF 1-4;  single family dwelling (270) | Residential; SF 1-4;  single family dwelling (272) |  |  | Residential; SF 1-4;  single family dwelling (281) |
|  | for parcels |  |  |  | Residential; |  | Residential; |  |  |  |  | Residential; | Residential; |  |
|  | with a CE |  |  |  | SF 1-4; |  | SF 1-4; |  |  |  |  | SF 1-4; | SF 1-4; |  |
|  | at some |  |  |  | single family |  | single family |  |  |  |  | single family | single family |  |
|  | time during |  |  |  | dwelling |  | dwelling |  |  |  |  | dwelling | dwelling |  |
|  | our |  |  |  | (221) |  | (231) |  |  |  |  | (274) | (277) |  |
|  | timeframe (count) |  |  |  |  |  |  |  |  |  |  |  |  |  |
|  |  | Vacant; Vacant Residentia  l (62) | Vacant; Vacant Ag (130) | Vacant; Vacant Ag (132) | Vacant; Vacant Ag (134) | Vacant; Vacant Ag (134) | Vacant; Vacant Ag (133) | Vacant; Vacant Ag (134) | Vacant; Vacant Ag (135) | Vacant; Vacant Ag (132) | Vacant; Vacant Ag (136) | Vacant; Vacant Ag (140) | Vacant; Vacant Ag (141) | Vacant; Vacant Ag (142) |
|  | LU types for those  parcels | Agricultur e; Crops  (47) | Agriculture  ; Crops  (53) | Agriculture  ; Crops (77) | Agriculture; Crops (90) | Agriculture  ; Crops  (128) | Agriculture; Crops (148) | Agriculture  ; Crops  (167) | Agriculture  ; Crops  (188) | Agriculture  ; Crops  (209) | Agriculture  ; Crops  (233) | Agriculture; Crops (275) | Agriculture; Crops (327) | Agriculture  ; Crops  (376) |

|  | with CEs in | Ag.; |  |  |  |  |  |  |  | Residential; SF 1-4;  single family dwelling (128) | Residential; SF 1-4;  single family dwelling (135) |  |  | Residential; SF 1-4;  single family dwelling (293) |
| --- | --- | --- | --- | --- | --- | --- | --- | --- | --- | --- | --- | --- | --- | --- |
|  | that year | Industrial |  |  |  |  |  |  |  |  |  |  |  |  |
|  | (count) | Ag.; |  |  |  |  |  |  |  |  |  |  |  |  |
|  |  | dairy |  |  |  |  |  |  |  |  |  | Residential; | Residential; |  |
|  |  | (with or | Vacant; | Vacant; | Vacant; | Vacant; | Vacant; | Vacant; | Vacant; |  |  | SF 1-4; | SF 1-4; |  |
|  |  | without | Vacant Ag | Vacant Ag | Vacant Ag | Vacant Ag | Vacant Ag | Vacant Ag | Vacant Ag |  |  | single family | single family |  |
|  |  | residence | (37) | (54) | (54) | (77) | (82) | (88) | (91) |  |  | dwelling | dwelling |  |
|  |  | or |  |  |  |  |  |  |  |  |  | (205) | (251) |  |
|  |  | manufactu |  |  |  |  |  |  |  |  |  |  |  |  |
|  |  | red home) |  |  |  |  |  |  |  |  |  |  |  |  |
|  |  | (4) |  |  |  |  |  |  |  |  |  |  |  |  |
|  |  | Residentia |  | Residential; | Residential; SF 1-4;  single family dwelling (30) | Residential; | Residential; SF 1-4;  single family dwelling (63) | Residential; | Residential; | Vacant; Vacant Ag (95) | Vacant; Vacant Ag (107) | Vacant; Vacant Ag (128) | Vacant; Vacant Ag (147) | Vacant; Vacant Ag (161) |
|  |  | l; SF 1-4; | Vacant; | SF 1-4; |  | SF 1-4; |  | SF 1-4; | SF 1-4; |  |  |  |  |  |
|  |  | single | Vacant | single |  | single |  | single | single |  |  |  |  |  |
|  |  | family | Residential | family |  | family |  | family | family |  |  |  |  |  |
|  |  | dwelling | (12) | dwelling |  | dwelling |  | dwelling | dwelling |  |  |  |  |  |
|  |  | (3) |  | (15) |  | (45) |  | (78) | (90) |  |  |  |  |  |
|  | Total # of | 63 | 141 | 205 | 260 | 359 | 416 | 456 | 506 | 589 | 642 | 850 | 1009 | 1167 |
|  | CE parcels |  |  |  |  |  |  |  |  |  |  |  |  |  |
|  | # of CEs |  |  |  |  |  |  |  |  |  |  |  |  |  |
|  | added by | 17 | 18 | 19 | 23 | 27 | 25 | 16 | 24 | 19 | 20 | 38 | 27 | 35 |
|  | Year |  |  |  |  |  |  |  |  |  |  |  |  |  |
|  | Avg. CE | 44.50 | 31.45 | 28.94 | 26.86 | 25.44 | 27.03 | 26.59 | 26.28 | 24.50 | 25.70 | 22.94 | 22.13 | 21.38 |
|  | parcel size |  |  |  |  |  |  |  |  |  |  |  |  |  |
|  | Median CE | 36.84 | 23.73 | 21.58 | 16.47 | 14.85 | 16.65 | 18.73 | 17.98 | 13.79 | 15.18 | 11.03 | 10.48 | 10.12 |
|  | parcel size |  |  |  |  |  |  |  |  |  |  |  |  |  |
|  | Min CE | 0.17 | 0.17 | 0.08 | 0.08 | 0.08 | 0.08 | 0.08 | 0.08 | 0.08 | 0.08 | 0.07 | 0.07 | 0.07 |
|  | parcel size |  |  |  |  |  |  |  |  |  |  |  |  |  |
|  | Max CE | 294.33 | 294.33 | 294.33 | 294.33 | 294.33 | 294.33 | 294.33 | 294.33 | 294.33 | 294.33 | 294.33 | 294.33 | 294.33 |
|  | parcel size |  |  |  |  |  |  |  |  |  |  |  |  |  |
|  | St’d | 44.16 | 35.76 | 33.06 | 32.42 | 30.13 | 34.21 | 32.58 | 31.56 | 30.94 | 34.04 | 31.54 | 30.49 | 29.30 |
|  | deviation |  |  |  |  |  |  |  |  |  |  |  |  |  |
| **All Counties** | LU types for parcels with a CE | Agricultur e; Crops (481) | Residential  ; SF 1-4;  single family dwelling  (370) | Residential; SF 1-4;  single family dwelling  (697) | Residential; SF 1-4;  single family dwelling (788) | Residential; SF 1-4;  single family dwelling  (1010) | Vacant; Vacant Residential (1010) | Vacant; Vacant Residential (1334) | Vacant; Vacant Residential (1540) | Vacant; Vacant Residential (1814) | Vacant; Vacant Residential (2002) | Vacant; Vacant Residential (2124) | Vacant; Vacant Residential (2198) | Vacant; Vacant Residential (2388) |
|  |  | Residentia l; SF 1-4;  single family dwelling  (335) |  | Vacant; Vacant Residential (412) | Vacant; Vacant Residential (515) | Vacant; Vacant Residential (820) |  | Residential; SF 1-4;  single family dwelling  (1030) | Residential; SF 1-4;  single family dwelling  (1163) | Residential; SF 1-4;  single family dwelling  (1221) | Residential; SF 1-4;  single family dwelling  (1295) |  |  | Residential; SF 1-4;  single family dwelling  (1182) |
|  | at some |  |  |  |  |  | Residential; |  |  |  |  | Residential; | Residential; |  |
|  | time during |  | Agriculture |  |  |  | SF 1-4; |  |  |  |  | SF 1-4; | SF 1-4; |  |
|  | our |  | ; Crops |  |  |  | single family |  |  |  |  | single family | single family |  |
|  | timeframe |  | (354) |  |  |  | dwelling |  |  |  |  | dwelling | dwelling |  |
|  | (count) |  |  |  |  |  | (995) |  |  |  |  | (1280) | (1253) |  |
|  |  | Ag. (232) | Ag. (272) | Ag. (348) | Agriculture; Crops (335) | Agriculture  ; Crops (335) | Agriculture; Crops (335) | Agriculture  ; Crops (336) | Agriculture  ; Crops (330) | Agriculture  ; Crops (330) | Agriculture  ; Crops (326) | Agriculture; Crops (319) | Residential;  Misc Res; HOA (364) | Residential;  Misc Res; HOA (386) |
|  | LU types  for those parcels | Ag.;  Crops (47) | Vacant;  Misc. Vacant; | Ag. (150) | Residential; SF 1-4;  single family | Vacant; Vacant | Vacant; Vacant | Vacant; Vacant | Vacant; Vacant | Vacant; Vacant | Vacant; Vacant | Vacant; Vacant | Vacant; Vacant | Vacant; Vacant |

|  | with CEs in |  | undevelopa |  | dwelling | Residential | Residential | Residential | Residential | Residential | Residential | Residential | Residential | Residential |
| --- | --- | --- | --- | --- | --- | --- | --- | --- | --- | --- | --- | --- | --- | --- |
|  | that year |  | ble (65) |  | (228) | (485) | (661) | (972) | (1126) | (1500) | (1772) | (1927) | (1990) | (2212) |
|  | (count) | Ag.; | Residential  ; SF 1-4;  single family  dwelling (56) | Residential; SF 1-4;  single family  dwelling (136) |  | Residential; SF 1-4;  single family  dwelling (383) | Residential; | Residential; SF 1-4;  single family  dwelling (623) | Residential; SF 1-4;  single family  dwelling (895) | Residential; SF 1-4;  single family  dwelling (1039) | Residential; SF 1-4;  single family  dwelling (1219) | Residential; | Residential; | Residential; SF 1-4;  single family  dwelling (1367) |
|  |  | Pasture; |  |  | Residential; |  | SF 1-4; |  |  |  |  | SF 1-4; | SF 1-4; |  |
|  |  | dry |  |  | SF 1-4 ; SF |  | single family |  |  |  |  | single family | single family |  |
|  |  | pasture |  |  | 1 – 5 (184) |  | dwelling |  |  |  |  | dwelling | dwelling |  |
|  |  | (24) |  |  |  |  | (508) |  |  |  |  | (1357) | (1382) |  |
|  |  | Ag.;  Crops; field crops (irrigated and dry)  (21) | Ag. (53) | Ag.;  Pasture; dry pasture (96) | Vacant; Vacant Residential (138) | Ag.;  Pasture; dry pasture (149) | Ag.; Pasture; dry pasture (174) | Residential (206) | Vacant; Misc Vacant;  10.0 Acres but < 35.0  Acres (243) | Vacant; Misc Vacant;  10.0 Acres but < 35.0  Acres (272) | Vacant; Misc Vacant;  10.0 Acres but < 35.0  Acres (272) | Vacant; Misc Vacant; 10.0 Acres but <  35.0 Acres (304) | Residential (342) | Ag.; Crops (376) |
|  | Total # of | 1247 | 1681 | 2711 | 2997 | 4073 | 4651 | 5339 | 6072 | 6754 | 7434 | 8313 | 8572 | 9066 |
|  | CE parcels |  |  |  |  |  |  |  |  |  |  |  |  |  |
|  | # of CEs |  |  |  |  |  |  |  |  |  |  |  |  |  |
|  | added by | 144 | 152 | 220 | 236 | 274 | 326 | 218 | 211 | 209 | 280 | 258 | 134 | 161 |
|  | Year |  |  |  |  |  |  |  |  |  |  |  |  |  |
|  | Avg. CE | 10.51 | 13.16 | 16.13 | 18.36 | 19.10 | 23.19 | 24.39 | 23.21 | 23.64 | 23.66 | 24.11 | 25.24 | 24.26 |
|  | parcel size |  |  |  |  |  |  |  |  |  |  |  |  |  |
|  | Median CE | 0.26 | 0.66 | 0.87 | 1.64 | 1.65 | 2.80 | 2.85 | 2.65 | 2.88 | 3.53 | 3.52 | 3.77 | 3.77 |
|  | parcel size |  |  |  |  |  |  |  |  |  |  |  |  |  |
|  | Min CE | 0.00 | 0.00 | 0.00 | 0.00 | 0.00 | 0.00 | 0.00 | 0.00 | 0.00 | 0.00 | 0.00 | 0.00 | 0.00 |
|  | parcel size |  |  |  |  |  |  |  |  |  |  |  |  |  |
|  | Max CE | 475.28 | 475.28 | 1280.52 | 1280.52 | 1280.52 | 3615.49 | 7692.61 | 7692.61 | 7692.61 | 7692.61 | 7692.61 | 7692.61 | 7692.61 |
|  | parcel size |  |  |  |  |  |  |  |  |  |  |  |  |  |
|  | St’d | 36.90 | 37.16 | 50.22 | 51.49 | 53.46 | 101.87 | 145.54 | 136.95 | 132.98 | 127.99 | 123.73 | 149.77 | 118.93 |
|  | deviation |  |  |  |  |  |  |  |  |  |  |  |  |  |
