# Supplementary material for: Unpacking conservation easements’ assessed land use designations and their implications for realizing biodiversity protection
Source: Conserv Sci Pract. Author manuscript; Available in PMC 2025 Jun 1. (PMC11675600; doi:10.1111/csp2.13130)
Supplement: Data S1 [file NIHMS1988779-supplement-Data_S1.docx]

# Supporting Information: Dataset Construction and Additional Results

**Dataset Construction, additional details**

The three criteria described in the article resulted in the original choice of California, Colorado, Massachusetts, Minnesota, Nebraska, and Virginia. However, after investigating data availability in Massachusetts, Nebraska, and then Michigan in lieu of Nebraska for Great Plains representation, we found that either the taxation data weren’t available at the county level and were inconsistent in record-keeping at the city levels, or the state did not have enough population growth (i.e., had negative growth in all possible counties) to meet our criteria. We substituted Montana for either Michigan or Nebraska, and New Jersey for Massachusetts (understanding that MA, MI, NJ, and NB all have state enabling laws with some linkage between CE placement and land use planning, creating public oversight). Again, we found that prohibitive cost, lack of digitization or county-level data amassing, paper or microfiche formatting, sheer lack of longevity in record-keeping, or low easements counts (less than 25 for our time period) meant that we had to substitute Pennsylvania for New Jersey in the Northeast, and South Carolina for Montana. Consequently, we lost the majority of the Great Plains representation and the balance of state-mandated land use planning concepts in CE placement in state representation. Our final states are: California, Colorado, Minnesota, Pennsylvania, South Carolina, and Virginia. Virginia is the only state with enabling legislation that includes a form of public oversight in CE placement.

We encountered similar ecoregional representation and data availability issues with some of the counties in our states of choice (i.e., Minnesota), which means that we do not have a Northern forests county in MN with the requisite CE data. But we were able to locate alternate counties to allow a two-county data collection process in this state. Effectively, we have had to choose new counties that are slightly less ideal but fit the criteria, principles, and of those, two (Douglas, MN, and Loudon, VA) still have limited tax valuation information electronically (with the remainder in hard copy or microfiched for a portion of the years of study (1997 – 2000)). Given the cost, time, and labor availability for digitization, we decided not to pursue those earlier years for the two counties.

# Master Land Use Coding Process, additional details

The mapping process illustrated in Appendix S1 corresponds to the following reasons; assumptions based on the land use type.

Residential

We reclassified the residential properties to create larger categories that would be comparable across counties. Where one county was more specific than another, we used the least specific category (e.g., rural residential home site in lieu of rural residential home site over 2 acres or rural residential home site with a single-family dwelling and a granny unit).

Retail and Commercial

Retail and commercial are also consolidated, but more problematic because one county assesses based on the kind of tenant in the specific land use, and another assesses based on the nature of the structure (i.e., the number of the stories) combined with kind of use. Several more detailed

categories had to be collapsed into a general one (e.g., service station). For instance, the “Office” general land use for Sacramento was collapsed based on the occupancy for most of the uses (e.g. bank, savings and loan, broadcasting radio and; or tv, etc.). Only the specific office land use distinguished occupancy, to reflect and maintain consistency across counties. Medical and dental offices were collapsed in one category for the same reason.

Industrial

We collapsed industrial (heavy and light) into general categories, which loses some of the detail in some counties.

Agriculture

We collapsed some of the irrigated farm properties into larger categories (i.e., premium varietals and vines and bush fruits into vineyard generally) to match other counties’ categories (i.e., grape vines)—as well as combined irrigated and dry orchards, and irrigated and dry vineyards. We collapsed irrigated field crops into field crops generally, and did not distinguish between combined uses (in terms of which is first, such as irrigated pasture and row crop versus row crop and irrigated pasture). We also collapsed dairy into one category (including their associated residences and mobile homes), and did the same for chicken and turkey farms, and horse ranches. We dropped transitional uses converting to vineyards for a county, instead placing that in miscellaneous agriculture, since this is such a specific use. We collapsed timber land into one category and did the same for hardwoods and chaparral.

Institutional Properties

We collapsed the church categories into general religious to match across counties, as well as collapsed religious building and its ancillary properties into one category. Schools are also collapsed into one category (private, parochial, special). We combined orphanages and homes for the handicapped in one county because there was no distinction in other counties.

Government Properties, Vacant Land, and Miscellaneous

We collapsed government properties by levels of government ownership into property or possessory interest for consistency. Vacant land uses are collapsed into the land use type and whether they have utilities (generally). Acreage isn’t distinguished because some counties don’t do so. For “Miscellaneous” we collapsed the character of use into the specific land use for consistency, and we collapsed unsecured uses into one category and included it in miscellaneous because the uses aren’t relevant to the work that we’re doing.

# Additional Results

Appendix S2 shows the one-way ANOVA results for the county CE parcel size means. Appendix S3 is a figure displaying the disaggregated trends in the land use designations for the parcels with a CE at some point in our timeframe across all of the counties. Some categories were aggregated because their counts were individually so low that they skewed the figure axes. These were:

- Agriculture: industrial, orchard, and vineyards
- Industrial: mixed, warehousing/storage, and general
- Institutional: institutional residential, misc., religious, medical, and schools
- Miscellaneous: exempt, land, and riparian
- Public/Gov’t & Utilities: city, county, federal, and state
- Public/Gov’t & Utilities: misc. gov’t property, special district utilities, and partially exempt
- Recreational: recreational, public, private, and miscellaneous
- Residential: lodging, mobile home/park, rural residential, MF – 5 or more, and misc.

Appendix S4 shows the more detailed descriptive statistics for each county, and the counties as a group. The datasets are structured for several statistical analysis approaches, whether from the perspective of the CE or the parcel. Consequently, the parcel identifiers may be replicated if there are multiple CEs on a parcel (or parts of several CEs), and the CE identifiers may be replicated if there are several parcels associated with a single CE. To accommodate, we examined the MLUDs for the parcels that contained a CE at any point in time during our time frame, but limited the analysis to the first time that a parcel identifier appeared. No parcel has more than 6 duplications in the datasets (which we reduced to 1 for that analysis). We also examined the MLUDs based on the CEs and when they were present on a parcel, in a given year. Our selection syntax counted the CEs that were present in that year and the previous years; we isolated the MLUDs of those associated parcels accordingly.

Appendix S5 shows the land use by parcel size for each of the individual codes across the counties, and their broader category is indicated by the leading digit.

Appendix S6 displays the consolidated LUD by mean, median, and standard deviation of parcel size for all counties, with the caveat that we did not include two outliers parcels with CEs in Greenville County, as these are CEs used to protect public drinking water and their size considerably skewed the general pattern.

Appendix S7 provides graphs of the first listed CE reason counts across all counties over time (1997 – 2009).

**Limitations**

It is also important to note the limitations associated with this work. First, we have not yet ascertained the level of naturalness on these CE parcels (Fouch et al., 2019). Additionally, our findings are unidimensional, meaning that our analysis shows only the CE reasons and the associated county LUD (pre- and post-CE placement). The CE may have been placed to contravene or effect the county’s LUD, or to prevent an even more intense land use conversion. And the CE may reflect the working nature of the land (e.g., agricultural or forestal), but we do not know the extent of the associated biodiversity preservation per Brunson and Huntsinger (2008). The CE may also stymie the LUD into more biologically friendly uses (e.g., the conservation subdivision).
